# EMG analysis across different tasks improves prevention screenings in diabetes: a cluster analysis approach

**DOI:** 10.1007/s11517-022-02559-3

**Published:** 2022-04-15

**Authors:** Weronika Piatkowska, Fabiola Spolaor, Annamaria Guiotto, Gabriella Guarneri, Angelo Avogaro, Zimi Sawacha

**Affiliations:** 1 Department of Information Engineering, University of Padova, Via Gradenigo 6B, 35131 Padova, Italy; 2 Department of Clinical Medicine and Metabolic Disease, University Polyclinic, Padova, Italy; 3 Department of Medicine, University of Padova, Padova, Italy

**Keywords:** Diabetes mellitus, Diabetic neuropathies, Stair climbing, Gait analysis, Electromyography, Clustering

## Abstract

The aim of this work was twofold: on one side to determine the most suitable parameters of surface electromyography (sEMG) to classify diabetic subjects with and without neuropathy and discriminate them from healthy controls and second to assess the role of the task acquired in the classification process. For this purpose 30 subjects were examined (10 controls, 10 diabetics with and 10 without neuropathy) whilst walking and stair ascending and descending. The electrical activity of six muscles was recorded bilaterally through a 16-channel sEMG system synchronised with a stereophotogrammetric system: Rectus Femoris, Gluteus Medius, Tibialis Anterior, Peroneus Longus, Gastrocnemius Lateralis and Extensor Digitorum. Spatiotemporal parameters of gait and stair climbing and the following sEMG parameters were extracted: signal envelope, activity duration, timing of activation and deactivation. A hierarchical clustering algorithm was applied to the whole set of parameters with different distances and linkage methods. Results showed that only by applying the Ward agglomerative hierarchical clustering (Hamming distance) to the all set of parameters extracted from both tasks, 5 well-separated clusters were obtained: cluster 3 included only DS subjects, cluster 2 and 4 only controls and cluster 1 and 5 only DNS subjects. This method could be used for planning rehabilitation treatments.

**Graphical abstract:**

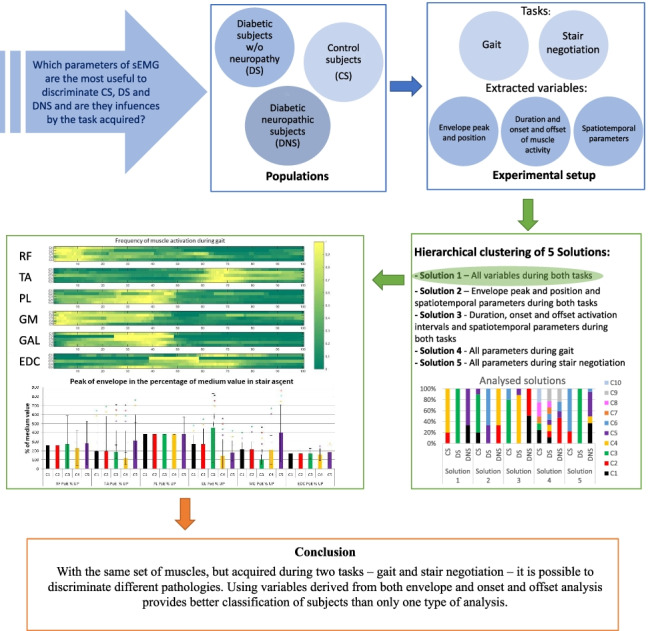

**Supplementary Information:**

The online version contains supplementary material available at 10.1007/s11517-022-02559-3.

## Introduction

Diabetes is one of the most widespread chronic diseases with the number of affected subjects increasing every year [1]. It causes many severe complications, amongst which the most common is diabetes peripheral neuropathy (DN). Up to 50% of all diabetics are affected by DN that may lead to the development of foot ulcers, loss of motor units in muscles and reduction of muscle volume [2, 3].

Changes in the neuromuscular system caused by DN were widely analysed by researchers in terms of kinematics, kinetics and surface electromyography (sEMG). The majority of these studies analysed muscle activity during gait or stair climbing in healthy (CS) and diabetic subjects with DN (DNS) and without neuropathy (DS).

Muscle functioning during gait was studied by Kwon [4], who detected an earlier activation of calf muscles in DNS, with the prolonged activity of Tibialis Anterior. Co-contraction of agonist and antagonist muscles at the ankle and knee joints was identified more often in DNS. Akashi [5] reported no differences in peak activation of muscles between CS and DNS; however, delayed activation of Vastus Lateralis and Lateral Gastrocnemius was observed in DNS with history of ulcers. Sawacha [6] detected early peak of activation of Rectus Femoris in both DS and DNS at the beginning of the stance phase, delay in activation of Gastrocnemius Lateralis during midstance and Rectus Femoris and Gluteus Medius during terminal swing in DS.

Muscle activity during stair negotiation was studied by Onodera [7], who indicated that during stair ascent (SA) the dorsiflexion of the ankle joint was reduced. During stair descent (SD) reduced plantar flexion of the ankle joint and reduced activity of Tibialis Anterior, during the phase of weight acceptance, were observed. Handsaker [8] reported delayed activation of knee and ankle extensor muscles in DNS, and knee extensors active longer during SA. Obtained results may be related to insufficient sensory feedback as mentioned by Spolaor [9], whose findings supported the hypothesis that changes caused by diabetes reduce the ability of subjects to perform some daily tasks. During a second phase of SA a delay in activity of Tibialis Anterior, Rectus Femoris and Extensor Digitorum Communis was detected in [9].

It should be mentioned that state of the art of sEMG analysis generally focus the attention on parameters such as the peak of the envelope occurrence and its value [5–7, 9, 10], and that the only work which reported results on the duration and onset and offset timing of the sEMG signal is the one of Handsaker et al. [8]. However the latter only analysed the activity of Vastus Lateralis and Gastrocnemius Medialis during gait and stair negotiation. Furthermore the results presented in the literature are not consistent, in some cases reduced or increased activity, and delayed and anticipated one was recorded on the same muscles (i.e. duration of Tibialis Anterior contraction in DNS; shortened in Sawacha et al. [6], Akashi et al. [5] and prolonged in Sacco et al. [10]). Understanding which parameters of sEMG are the most useful for discriminating between CS, DS and DNS could be useful to support clinicians in their clinical decision making process. For this purpose in the current study different sEMG parameters were extracted from the sEMG signals and a classification of subjects based on these parameters was proposed as alternative to the clinical driven classification [11].

Cluster analysis is a statistical technique that enables quantitative, objective identification of homogeneous groups in a population of interest [11–15]. For instance, the descriptive parameters which are placed within the same group are more similar to the ones placed in the other groups [12, 16]. Cluster analysis application on sEMG signals has not been widely used so far, yet it was successfully adopted as a tool for classifying subjects’ populations [17–20] and to guide medical treatment choices [21].

The aim of this work was twofold: on one side to find out which parameters of sEMG are the most useful to discriminate between CS, DS and DNS and second to verify if these parameters were influenced by the task acquired. To this extent, the present contribution presents a classification of CS, DS and DNS subjects driven by sEMG and spatiotemporal parameters derived from a dataset acquired during both gait and stair climbing. Hierarchical cluster analysis algorithms with different distances and linkage methods were adopted.

The main hypothesis is that the most appropriate classification will be obtained when analysing all the available parameters in both examined tasks. To successfully test this hypothesis, two secondary hypotheses were tested: (i) one of the examined tasks can discriminate subjects better than the other one and (ii) one category of parameters obtained by sEMG signal (i.e. onset-offset activation and activity duration vs peak of signal envelope) could better discriminate subjects than the other one.

## Methods

### Subjects

The sEMG measurements were performed at the Bioengineering of Movement Laboratory of the University of Padua and at the Padua University Clinics. DNS and DS were recruited amongst patients attending the Department of Metabolic Disease of the University of Padua. The control subjects (CS) were selected from hospital personnel. The inclusion criteria for DNS and DS incorporated type 1 or type 2 diabetes, ability to walk, no ulcers, no neurological disorders (other than DN), no orthopaedic problems or cardiovascular disease. All CS ought to be in good state of health, without diabetes, pathologies at the lower limbs and cardiovascular disease. All subjects gave written informed consent. The protocol for each study was approved by the Ethics Committee of the Padua University Clinics [6, 9]

Subjects’ numerosity was defined based on the power analysis [22] carried on previously published data [6] considering the value of the envelope peak as a variable; a number of 8 subjects was found to be sufficient for our analysis and 10 CS, 10 DS and 10 DNS were analysed compressively (see Table 1 for demographic parameters).

**Table 1:** Demographic data and the duration of performed tasks

	**CS**	ANOVA *p* < 0.05 *Z* test *p* < 0.05	**DS**	ANOVA *p* < 0.05 *Z* test *p* < 0.05	**DNS**	ANOVA *p* < 0.05 *Z* test *p* < 0.05
Total subjects	10	#	9	#	9	#
Gender (%)	30 male 70 female	#	80 male 20 female	#	67 male 33 female	#
Age (years)	61.2 (± 5)	#	62.0 (± 9)	#	62.0 (± 8)	#
Height (cm)	1.68 (± 0.13)	#	1.7 (± 0.03)	#	1.72 (± 0.07)	#
Weight (cm)	69.6 (± 17)	#	77 (± 7)	#	81 (± 15)	#
BMI (kg/m^2^)	24.4 (± 3)	#	26.5 (± 2)	#	27.9 (± 4)	#
HbA1c (mmol/ml)	/	#	7.1 (± 0.6)	#	7.9 (± 0.9)	#
Years of disease	/	#	12.6 (± 6)	#	23.2 (± 12)	#
Duration gait (ms)	1.07 (± 0.08)	CS vs DNS	1.08 (± 0.14)	#	1.11 (± 0.14)	CS vs DNS
Duration stance phase (ms)	0.64 (± 0.06)	#	0.65 (± 0.12)	#	0.67 (± 0.09)	#
Duration swing phase (ms)	0.43 (± 0.04)	#	0.43 (± 0.03)	#	0.43 (± 0.05)	#
Stance phase (%)	59.5 (± 2.3)	#	59.9 (± 3.7)	#	60.9 (± 2.2)	#
Swing phase (%)	40.5 (± 2.3)	#	40.1 (± 3.7)	#	39.1 (± 2.1)	#
Duration low step UP (ms)	0.62 (± 0.09)	CS vs DS and DNS	0.55 (± 0.10)	DS vs CS	0.61 (± 0.06)	DNS vs CD
Duration low step down (ms)	0.61 (± 0.10)	#	0.61 (± 0.09)	#	0.57 (± 0.08)	#
Duration high step up (ms)	0.69 (± 0.14)	CS vs DS	0.59 (± 0.09)	DS vs CS	0.69 (± 0.10)	#
Duration high step down (ms)	0.67 (± 0.16)	CS vs DS and DNS	0.56 (± 0.11)	DS vs CS	0.59 (± 0.07)	DNS vs CS

The statistically significant differences between different subject populations (CS, DS and DNS) are reported for each group.

*BMI* body mass index, *HbA1c* glycated haemoglobin.

### Clinical examination

Subjects’ feet were checked for skin lesions, bone deformities, ulcerations, signs of infection and previous amputations. Height (m) and weight (kg) were recorded and BMI (kg/m^2^) was calculated (see Table 1). Neurological evaluation included the assessment of symptoms and signs compatible with peripheral nerve dysfunction [23]. The Michigan Neuropathy Screening Instrument (MNSI) questionnaire [24] which evaluates motor and sensory symptoms was completed (based on MNSI score > 3). The physical examination consisted of (1) patellar and ankle reflexes; (2) assessment of muscle strength by ability to walk on heels, bilateral dorsal/plantar flexion of the feet, legs flexion/extension and abduction/adduction of forearms and fingers, all against resistance; (3) sensory testing carried out on the index finger and on the hallux (pin-prick with a disposable 25/7-mm needle), touch (10-g Semmens Weinstein monofilament, pathologic if no response on three out of 10 sites) and vibration perception threshold (VPT; 128-MHz tuning fork and Biothesiometer, pathologic if > 25 V); (4) pain sensitivity; (5) electroneurophysiological study; and (6) Index of Winsor (ankle-to-brachial index). The ulceration risk in 9 DNS was low and medium in 1 DNS [25]. Cardiovascular autonomic tests were also performed: they were considered positive if two or more tests were abnormal. HbA1c was also assessed. Each patient had at least an ophthalmologic examination, a urinary albumin-to-creatinine ratio, a carotid artery Doppler ultrasound, an ankle-brachial index determination and an electrocardiogram in the preceding 3 months. The presence of DN only was classified as low risk of ulceration, the presence of both DN and peripheral arterial diseases was classified as medium risk and the presence of DN and previous history of foot ulcers or amputation was classified as high risk. Clinical characteristics of the studied subjects are reported in Table 1.

### Instrumental evaluation

**Fig. 1:**
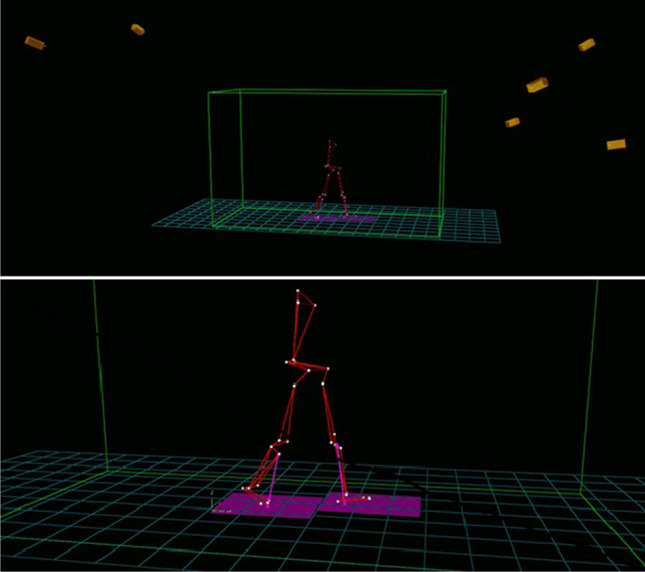
Experimental setup depicting subject performing analysed tasks

During SA and SD tasks and gait acquisitions, a BTS motion capture system (6 cameras, 60–120 Hz), 2 Bertec force plates (FP4060-10) and a 16-channel sEMG system (POCKETEMG, 16 channels, BTS Padova) were used. All instruments were synchronised. Setup is depicted in Fig. 1.

Muscles performing most essential roles in walking and stair negotiation were chosen [26] and amongst them the ones who revealed characteristic alterations in subjects with diabetes and DN [6, 9]. In particular the activity of Rectus Femoris (RF), Gluteus Medius (GM), Tibialis Anterior (TA), Gastrocnemius Lateralis (GAL), Peroneus Longus (PL) and Extensor Digitorum Communis (EXD) was recorded bilaterally at 1000 Hz during stair negotiation and gait. Sensors were positioned according to Blumenstein and Basmajian [27] and to Blanc and Dimanico [28] after appropriately cleaning and preparing the skin. Sensors were 24 mm of diameter and positioned 1 cm apart as described in Spolaor et al. [9]. sEMG was used for recording of muscle activity; motion analysis system and force plates were used for detecting spatiotemporal parameters during level walking and SA and SD. The IOR_gait [29] was adopted for the kinematic assessment.

Each subject performed 3 tasks — stair ascent, stair descent and gait. The following procedures were followed:

- ***Stair***: Patients were asked to ascend and descend a stair made of two steps 3 times at self-selected speed (2 steps, total height of 32 cm, each step 16 cm height × 81 cm wide × 28 cm depth, at the Bioengineering of Movement Lab (Department of Information Engineering of the University of Padova)); each step was positioned on top of a force plate and mechanical coupling assured with a double-sided tape according to [26].

Subjects were instructed to ascend the stairs, stop, turn around, wait for 1 s on the highest step and then to proceed with stair descent. A minimum of three SA and SD tasks were collected per subject.

- ***Gait***: Subjects were asked to walk barefoot at their preferred walking speed on the 8-m gait laboratory with two embedded force plates (Bertec, Canada) as in [9, 30]. A minimum of three walking trials per subject were collected.

### Data processing

Three trials per subject were considered in both gait and SA and SD analysis. The sEMG activity of 6 muscles was recorded bilaterally at 1000 Hz during both analysed activities.

Spatiotemporal parameters were defined by means of kinematic and kinetic data: during gait analysis the duration of gait cycle, swing phase and stance phase was estimated, as well as the percentage of the stance and the swing phases within the gait cycle by combining the trajectory of the marker applied on the calcaneus with the force plate data; in step negotiation analysis durations of low step up (the phase when the leading leg reaches the first step of the stair), high step up (the phase when the trailing leg rises up and takes place on the second step), low step down (the phase when the leading leg begins to descend from the stair and reaches the first step of the stair) and high step down (the phase when the trailing leg reaches the floor) were calculated as in Spolaor et al. [9] by combining the trajectory of the markers applied on the calcaneus, the 1st and the 5th metatarsal heads, with the force plate data.

From the recorded sEMG signals the following parameters were extracted:

- ***Envelope analysis***: Signals were band pass filtered between 10 and 450 Hz with a 5th-order Butterworth filter and full wave rectified. The envelope was computed by low-pass filtering the signals with a 4th-order Butterworth filter and a cutoff frequency of 5 Hz as in McFadyen and Winter [28]. The activations of the left and right muscles were analysed; for each gait cycle, the envelope of the signal was computed. The peak of each muscle sEMG activity as a percentage of the medium value in the cycle (PoE%), the timing of the peak with respect to the gait, SA and SD cycle (PPoE%) were defined, respectively, as in [31] and as in [9].
- ***Bonato-Knaflitz double threshold***: The signals were filtered using a filter removing heartbeat, notch filter for 50 Hz, band pass filtered with a double 5th-order Butterworth filter and full wave rectified. The cutoff frequencies varied from 5 to 15 Hz for high-pass filter, and between 450 and 495 Hz for low-pass filter. A double-threshold statistical detector proposed by Bonato [32] was applied for signal processing. The method [32] is based on the selection of the first threshold *ζ*, then by observing the chosen number of successive samples (*m*). Only if at least the chosen number of samples (*r*_0_), which is the second threshold in the observed interval, is above the first threshold, the signal is detected. The value of *ζ* is based on the level or the estimation of the background noise. All three parameters *ζ*, *r*_0_, and *m* are selected to minimise the false-alarm probability value and maximise the detection probability based on SNR value of each signal. Background noise is estimated for each signal based on the interval of subject’s static standing. Only activation intervals longer than 30 ms are accepted [32]. The values of duration of muscle activity and the onset and offset of muscle activity intervals, for each gait and stair negotiation cycle, were detected.

### Statistical analysis

Clustering is a multivariate technique aiming at classifying subjects based on the provided characteristics [12]. After a successful classification, formed clusters should be highly homogenous [12, 33]. Subjects’ similarities are compared to form groups including the most similar subjects from the input population. In the agglomerative hierarchical clustering each observation forms its own cluster, which is combined with the most similar one, using the chosen similarity measure. The process continues until all observations are contained in a single cluster [12].

In this contribution, hierarchical clustering with different linkage methods and distances was applied as follows: Euclidean, Manhattan and Hamming distances, together with Ward’s, complete and average linkage methods. The input data consisted of at least 90 vectors of data (3 cycles of each task for 30 subjects).

The parameters used for clustering were peak and position of the envelope within the task (i.e. gait cycle, SA and SD cycles), duration of each muscle activity, onset and offset of muscle activity and spatiotemporal parameters estimated on CS, DS and DNS during gait and stair negotiation. Clustering methods were implemented in Orange Canvas (orange.biolab.si).

In the present work 5 different sets of vectors were used as input to the cluster analysis:

- Solution 1 — Envelope peak and position, duration, onset and offset activation intervals and spatiotemporal parameters during gait and stair negotiation activities.
- Solution 2 — Envelope peak and position, spatiotemporal parameters during gait and stair negotiation activities.
- Solution 3 — Duration, onset, offset activation intervals and spatiotemporal parameters during gait and stair negotiation activities.
- Solution 4 — Envelope peak and position, duration, onset, offset activation intervals and spatiotemporal parameters during gait.
- Solution 5 — Envelope peak and position, duration, onset, offset activation intervals and spatiotemporal parameters during stair negotiation.

By considering that for each subject at least 3 repetitions of the same parameter were extracted for each task, one subject was assigned to a specific cluster only when at least 75% of the features were falling within the same cluster [11, 34]. This approach was used to avoid including possible outliers within the found groups.

No a priori hypotheses were formulated on the number of possible clusters, and visual inspection of dendrograms was adopted as selection criterion.

Furthermore a one-way ANOVA (SPSS, IMB Corp, Version 19.0) was performed across subjects classified into different clusters for each solution, on clinical and both sEMG and spatiotemporal parameters. A significance level of *p* < 0.05 was adopted for statistical analysis, using a Bonferroni correction when appropriate**.**

## Results

The data of 1 DNS and 1 DS were excluded from the study due to presence of artefacts in the signals; therefore, the data of 10 CS, 9 DS and 9 DNS were analysed during both tasks.

Age, years of disease and HbA1c showed no significant differences amongst groups. The hierarchical cluster analysis using Ward’s agglomerative linkage and Hamming distance considering either all of the parameters or their subset led to the definition of five to ten separate clusters (C1–C10): five clusters when considering all the parameters (Solution 1), ten clusters when considering all the parameters during gait task (Solution 4) and six clusters in other cases (Solutions 2, 3 and 5).

Within the Solution 5 six clusters were obtained: all DS were classified into a single cluster C3, and CS were divided into two clusters C2 (including only CS and they represented the 22% of CS population) and C6 (including CS and DS, with the 78% of CS mixed with the 6% of DNS); DNS were classified into 3 different clusters, C1 (37% of the sample), C4 (12% of the sample) and C5 (45% of the sample) (Fig. 1).

**Fig. 2:**
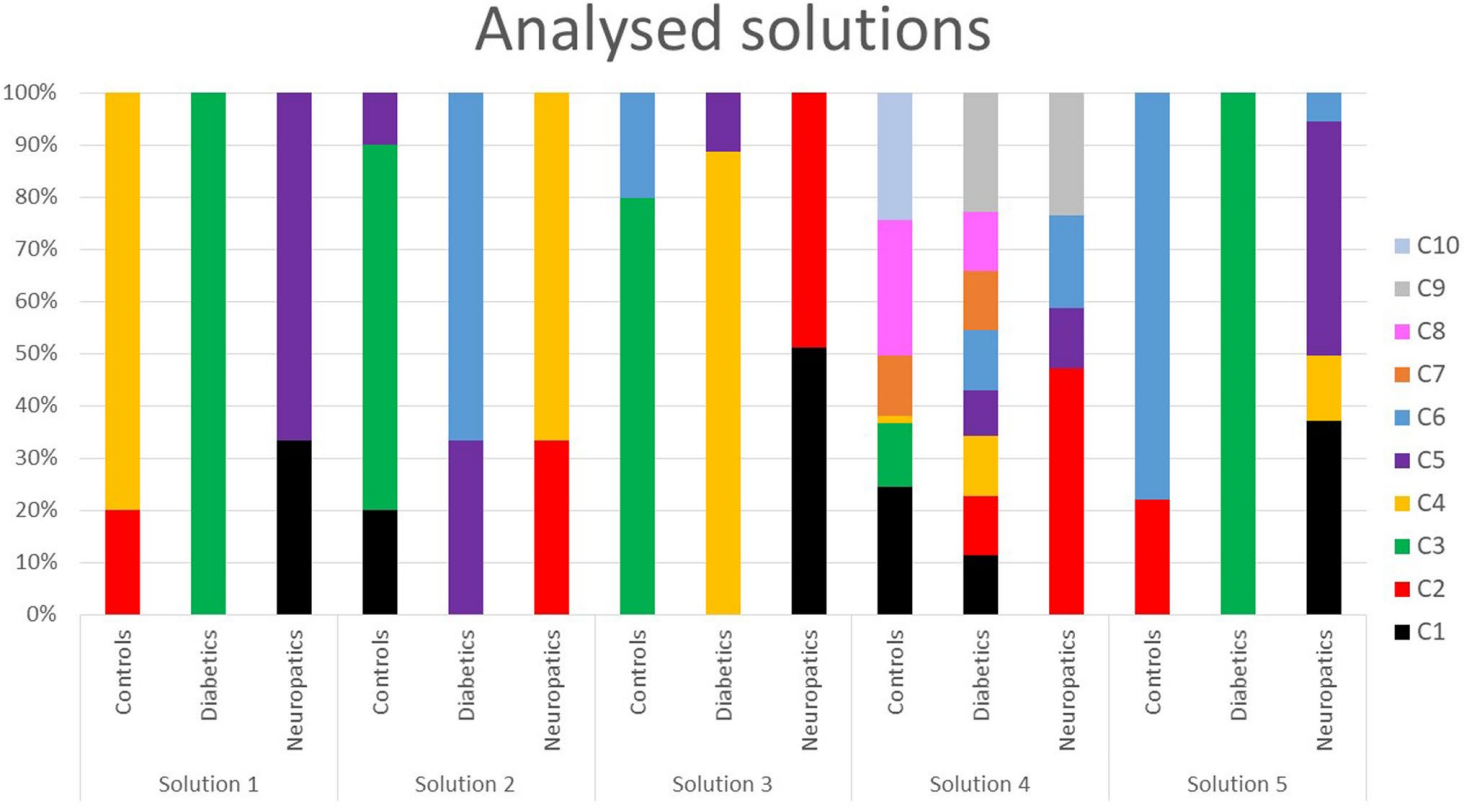
Distributions of percentage of CS, DS and DNS in clusters in all analysed solutions

Within the Solution 4 ten clusters were obtained: all groups of subjects were divided into multiple clusters; the majority of which were heterogeneous. CS were classified into 6 different cluster; amongst them, cluster C3 and C10 were homogenous. DS were classified into 8 different clusters; in half of them subjects were mixed with CS and in the other half with DNS. DNS were classified into 4 different clusters that included also DS (Fig. 2).

Within the Solution 3 six homogenous clusters were formed: all groups of subjects were classified into two distinct clusters. CS formed C3 and C6 (80% and 20% of CS, respectively); 89% of DS were included in C4 and 11% in C5, 51% of DNS were classified into C1 and 49% into C2 (Fig. 2).

Within the Solution 2 six clusters were obtained: CS were classified into 3 different clusters. C1 and C3 were homogenous and included 20% and 70% of subjects, respectively; C5 included 10% of CS mixed with 33% of DS; and the remaining 67% of DS formed C6. DNS were divided into two clusters; C2 included 33% of DNS and C4 included 67% of DNS (Fig. 2).

All the above solutions (from 2 to 5) were discarded (see Supplementary Material for a more detailed description).

**Fig. 3:**
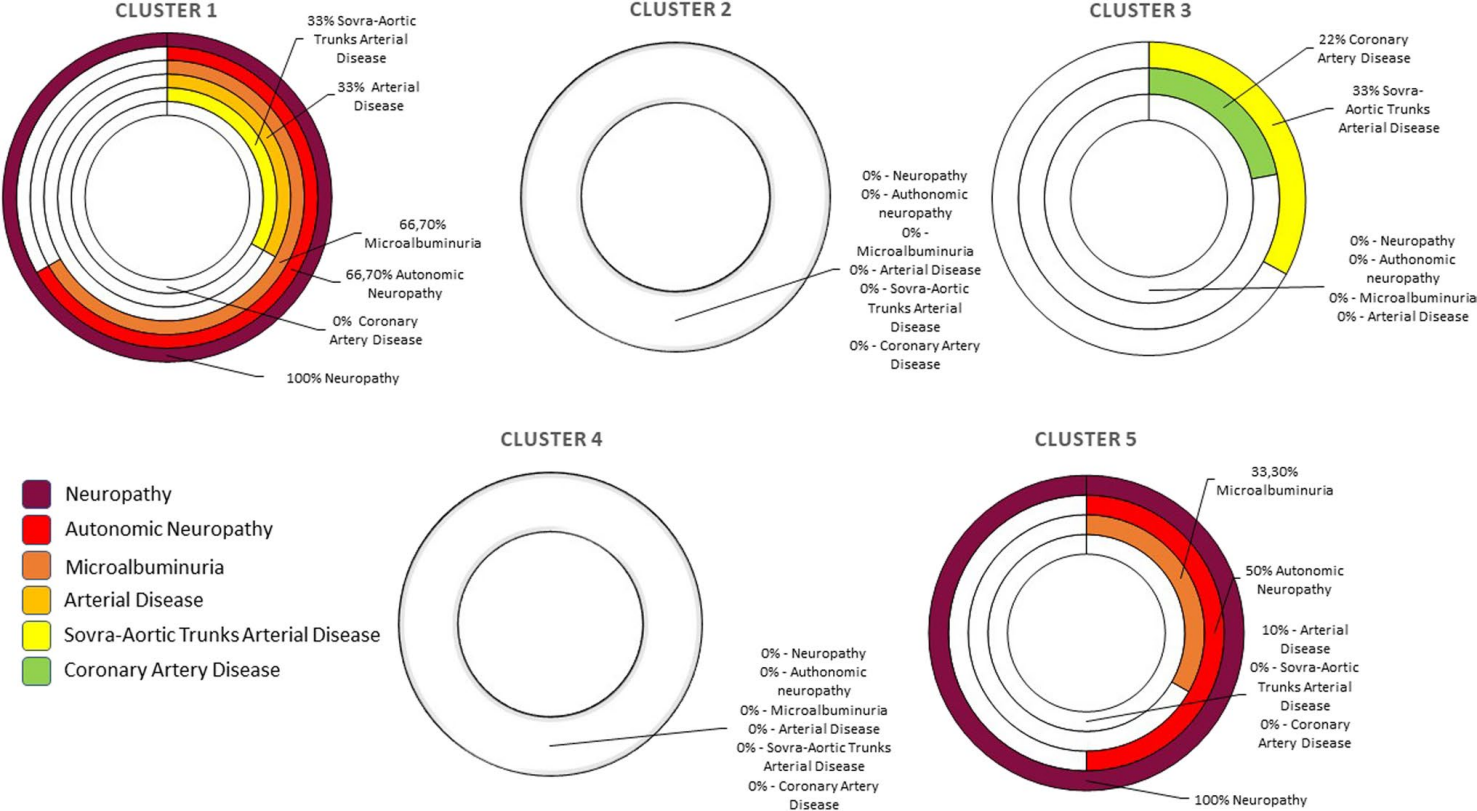
The clinical characteristics of subjects within clusters found in Solution 1: Neuropathy – brown, Autonomic Neuropathy – red, Microalbuminuria – darg orange, Arterial Disease – bright orange, Sovra-Aortic Trunks Arterial Disease – yellow, Coronary Artery Disease – green. Colours refer to the online version of the figure

Only Solution 1, driven by all the parameters during both tasks, was considered as providing clinically meaningful clusters: all DS were classified into a single cluster C3; CS and DNS were divided in two clusters, respectively (CS — 20% in C2 and 80% in C4, DNS — 33% in C1 and 67% in C5). Each cluster included data of a single population (Fig. 3).

**Fig. 4:**
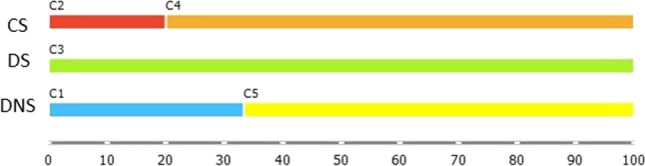
Distribution of percentage of CS, DS and DNS in clusters in Solution 1

The clinical characteristics of subjects within clusters found in the Solution 1 were reported in Fig. 4, where we observe that C1 and C5 displayed similar percentage of subjects affected by Autonomic Neuropathy (67% of subjects in C1 and 50% of subjects in C5) and differed in terms of presence of Sovra-Aortic Trunks Arterial disease, Arterial disease (reported only in subjects from C1). Furthermore a higher percentage of subjects affected by Microalbuminuria was recorded in C1 (67% of subjects in C1, 33% of subjects in C5).

The demographic data, spatiotemporal gait and SA/SD parameters within the clusters found in the Solution 1 are presented in Table 2. Statistically significant differences (*p* < 0.05) in the demographic parameters amongst the clusters were observed in weight (clusters C4 and C1, C2) and BMI (clusters C1 and C4). In terms of gait spatiotemporal parameters, subjects from C5 displayed the following statistically significant differences: longer duration of the gait cycle in comparison with all the other clusters; longer stance phase in C5 with respect to C2, C3 and C4; and longer duration of the swing phase with respect to C1. The latter was also detected in C4 with respect to C2. It is worth mentioning that even though C2 and C4 included only CS and C1 and C5 only DNS, the differences within the same population of subjects determined the association with different clusters. The analysis of the time parameters during stair negotiation revealed that a significantly longer low step up and high step down duration was revealed in subjects from C2; a longer low step down duration was observed in subjects from C1, and a longer high step up duration was detected in subjects from C1, C3 and C4.

**Table 2:** Demographic data and the duration of performed tasks

	**C1**	ANOVA *p* < 0.05 *Z* test *p* < 0.05	**C2**	ANOVA *p* < 0.05 *Z* test *p* < 0.05	**C3**	ANOVA *p* < 0.05 *Z* test *p* < 0.05	**C4**	ANOVA *p* < 0.05 *Z* test *p* < 0.05	**C5**	ANOVA *p* < 0.05 *Z* test *p* < 0.05
Total subjects	3	#	2	#	9	#	8	#	6	#
Age (years)	67.0 (± 7.8)	#	56.5 (± 9.2)	#	61.9 (± 9.0)	#	62.4 (± 3.6)	#	59.3 (± 7.2)	#
Height (cm)	1.75 (± 0.09)	#	1.87 (± 0.0)	#	1.71 (± 0.03)	#	1.65 (± 0.11)	#	1.70 (± 0.07)	#
Weight (cm)	92.5 (± 9.4)	C1 vs C4	105.0 (± 0.0)	C2 vs C4	77.4 (± 7.1)	#	65.1 (± 11.8)	C4 vs C1,C2	75.2 (± 14.6)	#
BMI (kg/m^2^)	30.1 (± 1.2)	C1 vs C4	30.0 (± 0.0)	#	26.5 (± 2.0)	#	23.7 (± 2.1)	C4 vs C1	26.8 (± 4.7)	#
HbA1c (mmol/ml)	8.6 (± 0.5)	#	/	#	7.1 (± 0.2)	#	/	#	7.6 (± 0.3)	#
Years of disease	21.3 (± 10.1)	#	/	#	12.6 (± 6.4)	#	/	#	24.2 (± 13.7)	#
Duration gait (ms)	1.05 (± 0.09)	C1 vs C5	1.01 (± 0.08)	C2 vs C3,C4 and C5	1.08 (± 0.14)	C3 vs C2 and C5	1.09 (± 0.08)	C4 vs C2 and C5	1.13 (± 0.15)	C5 vs C1, C2, C3 and C4
Duration stance phase (ms)	0.65 (± 0.08)	#	0.61 (± 0.06)	C2 vs C5	0.65 (± 0.12)	C3 vs C5	0.65 (± 0.06)	C4 vs C5	0.68 (± 0.10)	C5 vs C2, C3 and C4
Duration swing phase (ms)	0.39 (± 0.03)	C1 vs C3, C4 and C5	0.4 (± 0.02)	C2 vs C3, C4 and C5	0.43 (± 0.03)	C3 vs C1, C2, C4 and C5	0.44 (± 0.04)	C4 vs C1, C2 and C3	0.45 (± 0.06)	C5 vs C1, C2 and C3
Stance phase (%)	62.1% (± 2.4)	C1 vs C2, C3, C4 and C5	60.2% (± 1.8)	C2 vs C1	59.9% (± 3.7)	C3 vs C1, C2, C4 and C5	59.3% (± 2.4)	C4 vs C1 and C5	60.3% (± 1.7)	C5 vs C1 and C4
Swing phase (%)	37.9% (± 2.4)	C1 vs C2, C3, C4 and C5	39.8% (± 1.76)	C2 vs C1	40.1% (± 3.7)	C3 vs C1	40.7% (± 2.4)	C4 vs C1 and C5	39.7% (± 1.7)	C5 vs C1 and C4
Duration low step UP (ms)	0.6 (± 0)	C1 vs C2 and C3	0.7 (± 0.12)	C2 vs C1, C3, C4 and C5	0.55 (± 0.11)	C3 vs C1, C2, C4 and C5	0.61 (± 0.07)	C4 vs C2 and C3	0.62 (± 0.08)	C5 vs C2 and C3
Duration low step down (ms)	0.58(± 0)	C1 vs C2	0.64 (± 0.09)	C2 vs C1, C3 and C5	0.56 (± 0.09)	C3 vs C4 and C5	0.61 (± 0.11)	C4 vs C3 and C5	0.56 (± 0.10)	C5 vs C2 and C4
Duration high step up (ms)	0.66 (± 0)	C1 vs C2 and C3	0.76 (± 0.12)	C2 vs C1, C3 and C4	0.59 (± 0.09)	C3 vs C1, C2, C4 and C5	0.67 (± 0.14)	C4 vs C2 and C3	0.71 (± 0.12)	C5 vs C3
Duration high step down (ms)	0.61 (± 0)	C1 vs C2	0.73 (± 0.09)	C2 vs C1, C2 vs C19)	0.56 (± 0.10)	C3 vs C1, C3 vs C4 and C5	0.65 (± 0.18)	C4 vs C2 and C3	0.58 (± 0.09)	C5 vs C2 and C4

The statistically significant differences between clusters obtained in Solution 1 (C1–C5) are reported for each group.

*BM* body mass index, *HbA1c* glycated haemoglobin.

**Fig. 5:**
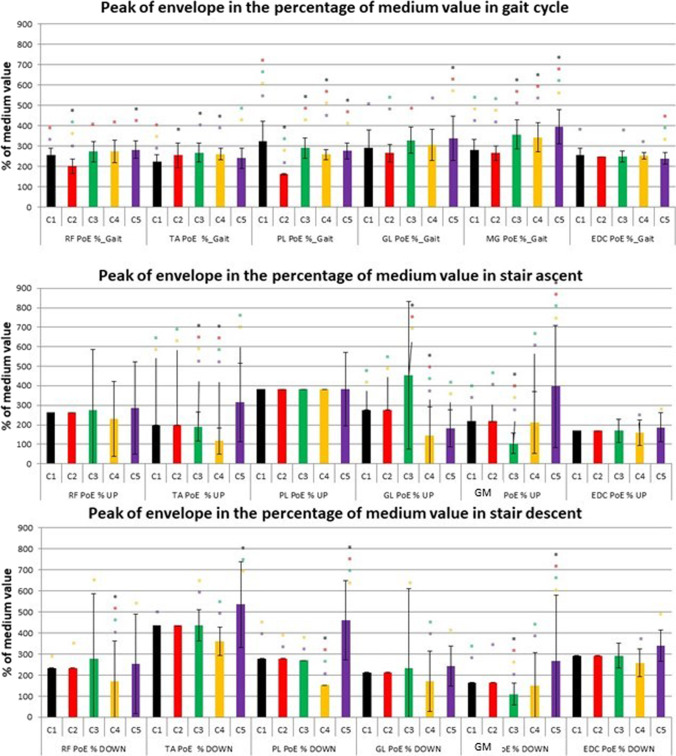
Peak of envelope in the percentage of medium value during each task for clusters found in Solution 1: C1 – black, C2 – red, C3 – green, C4 – yellow, C5 – violet. Statistically significant difference (*p* < 0.05) between clusters is signed with asterisk in the colour of cluster with respect to which a significant difference was found. Colours refer to the online version of the figure

**Fig. 6:**
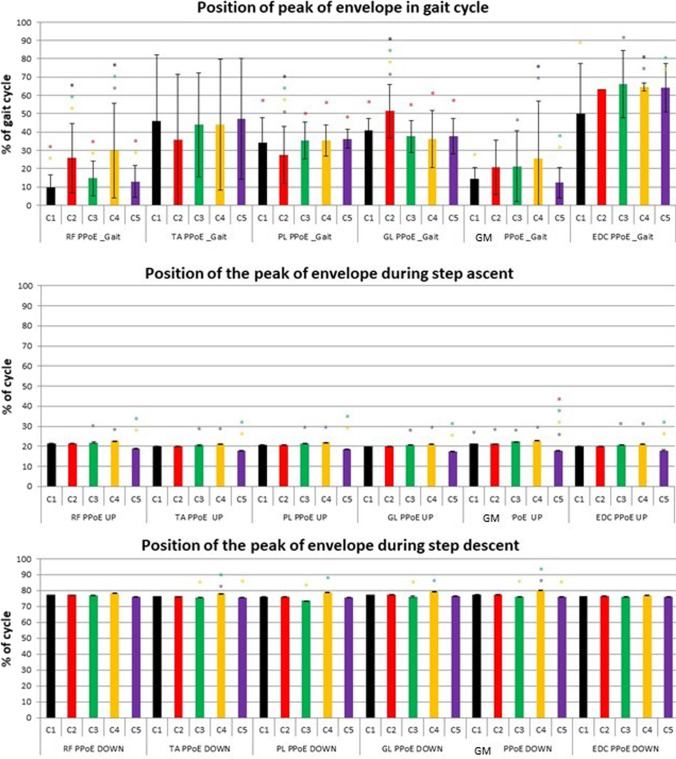
Position of the peak of envelope during each task for Solution 1: C1 – black, C2 – red, C3 – green, C4 – yellow, C5 – violet. Statistically significant difference (*p* < 0.05) between clusters is signed with asterisk in the colour of cluster with respect to which a significant difference was found. Colours refer to the online version of the figure

**Fig. 7:**
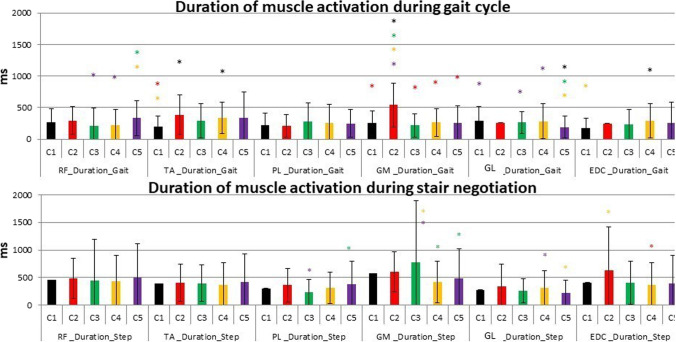
Duration of contraction in percentage of task cycle for Solution 1: C1 – black, C2 – red, C3 – green, C4 – yellow, C5 – violet. Statistically significant difference (*p* < 0.05) between clusters is signed with asterisk in the colour of cluster with respect to which a significant difference was found. Colours refer to the online version of the figure

**Fig. 8:**
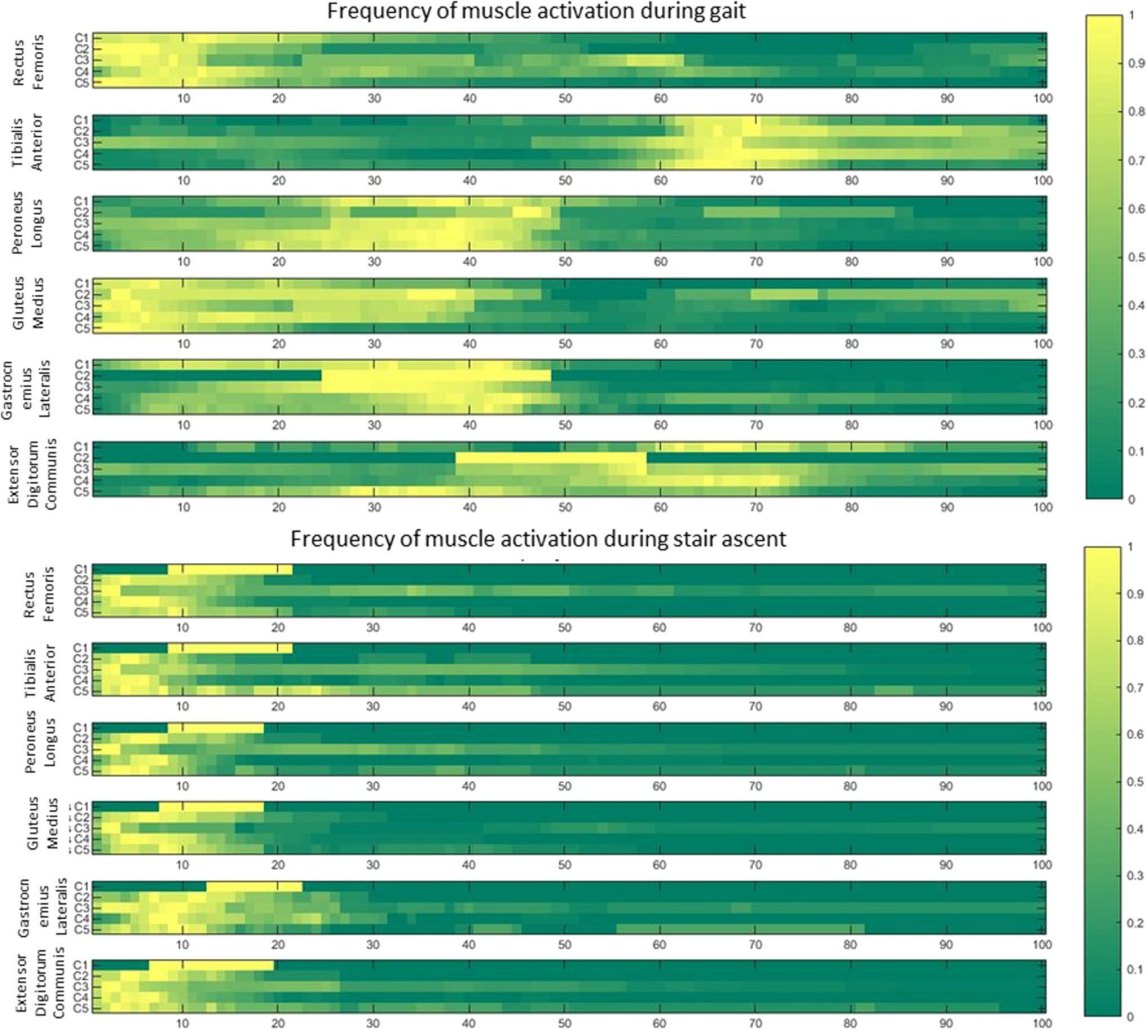
Frequency of muscle activation and deactivation in analysed muscles for clusters found in Solution 1: horizontal bars are coloured coded as in [32], according to the number of subjects in which a muscle activity at each percentage of gait cycle is observed; yellow: muscle activity is detected in all subjects; and dark green: muscle is not detected in any subject. Colours refer to the online version of the figure

The sEMG characteristics of each cluster are shown in Figs. 5, 6, 7 and 8. Results were organised as follows. First we introduce the differences amongst the clusters with respect to the sEMG parameters, and secondly, we describe how these parameters distributed the 3 cohorts of subjects within each cluster.

When considering the different tasks the differences observed in terms of sEMG were listed below referred, respectively, to gait and stair:

- Gait. In terms of the peak of the envelope the main statistically significant differences were the following: in RF, a decreased value in C2 with respect to all the other clusters; in PL, an increased value in C1 and a decreased value in C2 with respect to all the other clusters; and in GM, an increased value in C5 with respect to all the other clusters.

In terms of occurrence of the peak of the envelope the main statistically significant differences were detected in C2 and C4 with respect to all the other clusters: in RF, a delayed peak of activity (in both C2 and C4); in PL, an earlier peak of activity (in C2); and in GL, a delayed peak of activity (in C2).

In terms of duration of muscle activity the main statistically significant differences were the following: in RF, a longer duration in C5 with respect to C3 and C4; in TA, a shorter duration in C1 with respect to C2 and C4; in GM, a longer duration in C2 with respect to all the other clusters; and in GL, a shorter duration in C5 with respect to C1, C3 and C4.

In terms of muscle activation timing the main statistically significant differences were the following: in RF and GM, an earlier offset in C1, C3 and C5 during stance phase and a lack of muscle activation in C1 and C5 during terminal swing; in TA, an earlier offset of activity in C1, C3 and C5; in PL, an earlier activation onset in C3 and C5; and in GL, an earlier onset of activation in C1 and C3.

- Stair ascent. In terms of the peak of the envelope the main statistically significant differences were the following: in TA, a decreased value in C3 and C4 with respect to all the other clusters; in GL, an increased value in C3 and a decreased value in C4 with respect to all the other clusters; and in GM, an increased value in C5 and a decreased value in C3 with respect to all the other clusters.

In terms of the occurrence of the peak of the envelope the main statistically significant differences were the following: in GM, an earlier position in C5 with respect to all the other clusters and an earlier position in all the other muscles in C5 with respect to C3 and C4.

In terms of duration of muscle activity a significantly longer duration of GM was detected in C3 with respect to C4 and C5.

In terms of muscle activation timing a significantly delayed activation onset was detected in C1 in all the analysed muscles.

- Stair descent. In terms of the peak of the envelope the main statistically significant differences were the following: in RF, a decreased value in C2 with respect to all the other clusters; in PL, an increased value in C5 and a decreased value in C4 with respect to all the other clusters; and in GM, an increased value in C5 and a decreased value in C3 with respect to all the other clusters.

In terms of the occurrence of the peak of the envelope the main statistically significant differences were the following: in TA and GM, a later position in C4 with respect to C3 and C5 and in PL and GL, a later position in C4 with respect to C3.

When considering the three cohorts of subjects, CS were classified into two different groups; even though this distinction was not supported by statistical differences in the demographic data, it can be found in the spatiotemporal parameters and sEMG characteristics: C2 revealed shorter gait cycle, stair ascent and descent phases than C4. In terms of sEMG C4 presented a longer duration of GM activity, an earlier activation of PL and a delayed onset of RF and EDC during gait in comparison with C2. Moreover, in C2 lower values of the peak of the envelope were observed during gait in all analysed muscles, whilst higher values of the peak of the envelope were observed during stair negotiation.

All DS were classified into a single cluster (C3), whilst DNS were divided into two groups (C1 and C5), which differed in terms of muscle activity of PL, EDC, GM and GL. For instance, during gait subjects from C5 displayed an earlier activation of PL and EDC, delayed activation of GM and delayed and shortened activation of GL. Furthermore, the value of the peak of the envelope was higher in all the muscles except from PL. Oppositely, during the stair ascent all muscles displayed a delayed onset of muscle activity.

## Discussion

The aim of our study was to answer the question: which task and set of sEMG parameters is more suitable to provide clear guidance to people in charge of preventing diabetic foot? First of all we hypothesised that we could classify subjects with diabetes and diabetic neuropathy based only on sEMG parameters and secondly, that the most clinically appropriate classification would have been obtained when analysing a large set of sEMG parameters (i.e. intensity and timing related parameters) during different tasks characterizing daily living activities. Our results confirmed these hypotheses. Hierarchical clustering was employed using different distances and linkages (i.e. Euclidean, Manhattan and Hamming distances with Ward’s, complete and average linkage methods) and the best results were obtained when applying Ward’s agglomerative linkage with Hamming distance to the whole set of features extracted from two tasks, namely gait and stair ascending (see Fig. 2).

The results obtained with the approaches that adopted only a partial set of features did not lead to well-separated clusters and aggregated subjects belonging to different clinical groups or formed very small clusters (see Supplementary Material). This suggests that muscular alterations derived from diabetes and its complications are too complex to be fully described with a few parameters retrieved within a single task. In our work sEMG activity was used to drive the diabetic subject classification on parameters extracted from two tasks.

.Results showed that the data of CS subjects were classified into 2 different clusters (C2 and C4) including only healthy subjects, and this result is in accordance with literature investigating variability in normal gait patterns [17, 18, 34]. Activation and deactivation patterns of RF, GM and TA observed in both C2 and C4 were in line with the normal activation pattern of these muscles reported by Benedetti et al. [34]. It is worth mentioning that these two groups were different in terms of spatiotemporal parameters, and that subjects from C2 seemed to be more stable during monopodalic support [9]. All DS were classified into a single cluster (C3) and this suggests that all examined subjects presented a similar muscle activation pattern during both tasks.

Differently from DS, DNS were divided into two groups (C1 and C5): subjects classified into C1 performed stair negotiation tasks faster than subjects grouped in C5, which suggests the presence of a decreased stability during monopodalic support [9]. The earlier onset of muscle activity during stair ascent detected in the same cluster supports previous findings reporting that DNS are unable to efficiently control their weight bearing whilst stair ascending [8, 9, 35]. The lower values of GM during stair ascent and of TA, PL and GM during stair descent in subjects from C1 find agreement with the sensorimotor disturbances connected with DN [8, 9]. Furthermore, it is worth noticing that this cluster included subjects diagnosed with microalbuminuria and peripheral arterial disease. Both clusters including DNS were characterised by an earlier peak of the envelope registered in GM and a shorter duration of its contraction differently from the clusters including only CS, thus suggesting that DNS try to cope with the ankle rigidity [6], the reduced stabilisation and the decreased joint mobility of the hip [36, 37]. The earlier position of the peak of the envelope and the shorter activity recorded in GL finds agreement with what is observed by [4] about a reduced stabilisation of the knee in DNS. The shorter duration and the earlier onset of TA activity are also in accordance with the literature [7, 10], suggesting an inappropriate foot rollover during the stance phase in DNS. Furthermore, the delayed position of the peak of the envelope detected in PL accompanied by shorter (but non-significant) activation, delayed both onset and offset of the EDC, suggests the presence of a reduced ankle mobility and foot deformities in DNS. When considering DNS subjects the ones included in C1 seem to be more affected by muscular alterations associated with insufficient sensory feedback [8] than subjects from C5.

Some common alterations were detected in clusters including both DS and DNS (C1, C3, C5), such as the early position of peak of the envelope in RF during gait, which is in accordance with results presented in [6] and might be related to an attempt to prepare the leg for the contact with the ground during the heel strike or to decelerate the flexion of the hip and the extension of the knee during loading response. This finds agreement with the reduction of the hip mobility which characterises DNS as reported in the literature [6, 37–39].

Certain limitations exist for this study. Only the onset and offset and duration parameters extracted from the stair ascent phase of the stair negotiation were used, as muscle alterations highlighted with these parameters were similar in both phases. However, sEMG parameters associated with the envelope analysis extracted from both phases were used. Further studies should employ a larger sample size, and include other parameters (i.e. frequency parameters of sEMG signal). The lack of the application of automation techniques to optimise features used for classification is a limitation of the study; however, it should be taken into account that we aimed to investigate not only which set of sEMG parameters, but also which task would allow the best classification of diabetic and neuropathic subjects with respect to controls. Nevertheless, in many high-dimensional datasets the patterns captured by the PCA are those separating different subgroups from hierarchical clustering, thus leading to similar results [40]. Feature optimisation can be a subject of future investigation. Nonetheless, the large number of parameters extracted on a small number of subjects was in line with the state of the art describing application of data mining methods for the classification of gait parameters [19, 41–43]. Even though some of the present findings are contradictory with the current literature [9], it should be mentioned that previous studies compared subjects based on the diagnosis of DN, whilst the current study compared subjects based on the clustering results.

Overall, the alterations detected at the level of the muscular activity in all the DS and DNS could be used to plan dedicated physical activity protocols in order to improve ankle joint function and any associated alterations [44].

## Conclusions

Within the literature there is no agreement on which muscles and which task provide the most meaningful information about the muscular alterations caused by diabetes and its complications [5, 6, 10]. Our results suggest that with the same set of muscles, but acquired during two tasks — gait and stair negotiation — it is possible to discriminate between diabetic and neuropathic subjects and each of them from healthy controls. Moreover, by using parameters derived from both envelope and onset and offset analyses, a better classification of subjects is provided. In particular, the classification of DNS into two different clusters finds agreement with previous studies that detected different patterns in this population at the level of joint kinematics [11], kinetics, and plantar pressure [15, 43]. These results can be adopted on one hand as a support for clinical decision making, on the other one to plan studies aiming at detecting differences between diabetic subjects and other cohorts. It is worth mentioning that combining data from two types of sEMG analysis and investigating two tasks rather than only gait can substantially improve our understanding of the muscular alterations caused by diabetes and its complications.

## Supplementary Information

Below is the link to the electronic supplementary material.

Supplementary file1 (DOCX 678 KB)
