# Supplementary material for: EMG analysis across different tasks improves prevention screenings in diabetes: a cluster analysis approach
Source: Med Biol Eng Comput. 2022 Apr 15;60(6):1659–73. doi: 10.1007/s11517-022-02559-3 (PMC9079040; doi:10.1007/s11517-022-02559-3)
Supplement: Supplementary file 1 — Supplementary file1 (DOCX 678 KB) [file 11517_2022_2559_MOESM1_ESM.docx]

The appendix contains data of the discarded solutions analysed during the research described in the article. The discarded solutions are listed below:

- Solution 2 – Envelope peak and position; spatiotemporal parameters during gait and stair negotiation activities
- Solution 3 - Duration, onset and offset activation intervals and spatiotemporal parameters during gait and stair negotiation activities
- Solution 4 - Envelope peak and position, duration and onset and offset activation intervals and spatiotemporal parameters during gait
- Solution 5 - Envelope peak and position, duration and onset and offset activation intervals and spatiotemporal parameters during stair negotiation

The classification of subjects into clusters in all discarded solutions is presented in Fig. 1.


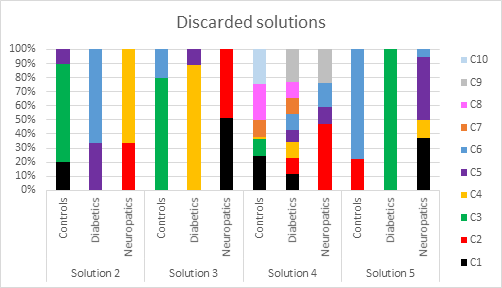


Figure 1. Distributions of percentage of CS, DS and DNS in clusters in discarded solutions.

Classification of subjects using data from Solution 2 - envelope parameters only during both tasks – led to the definition of 6 clusters. DNS were well separated from other groups – all subjects were classified into 2 homogenous clusters. CS were classified into 3 distinct clusters – 2 of them were homogenous and one was heterogeneous as it contained also DS. The latter cluster consisted of 10% of CS population and 33% of DS population. The remaining part of DS was classified into homogenous cluster C6. The distribution of subjects from different populations in clusters is presented in Figure 2. This solution was discarded due to the presence of a heterogeneous cluster.


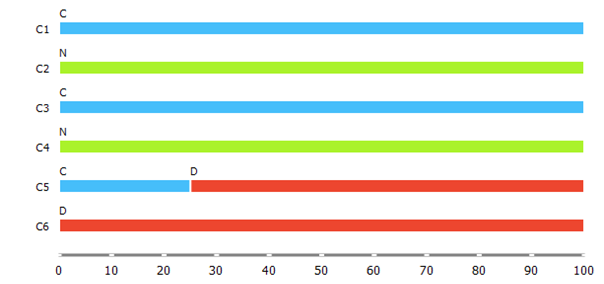


Figure 2. Distributions of percentage of CS, DS and DNS in clusters in Solution 2.

In the Solution 3 onset and offset of muscle activation and duration of muscle activation during both tasks were analysed. Each population was classified into two homogenous clusters. CS formed clusters with distribution of subjects C3 - 80% and C6 - 20%. The distribution of CS was the same as in the selected solution. The same subjects were classified together. DNS were classified into clusters with distributions C1 - 51% and C2 - 49%. DS were classified into clusters with distribution C4 - 89% and C5 - 11%. The only statistically significant difference in the EMG parameters between C4 and C5 was in the onset and offset of muscle activation during stair negotiation – muscular activation initiation and cessation was detected earlier in subjects from C5. The distribution of subjects from different populations in clusters is presented in Figure 3.

Solution 3 was the second best solution. It was discarded due to the fact that adding envelope parameters to the input set of variables allowed classification of all DS into a single cluster.


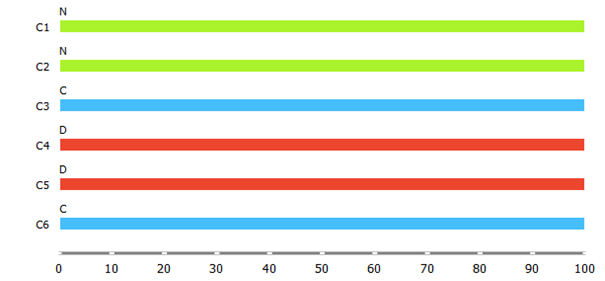


Figure 3. Distributions of percentage of CS, DS and DNS in clusters in Solution 3.

Solution 4 presented the most heterogeneous results, 10 clusters were formed, only 2 of them were homogenous. All populations were classified into multiple clusters – CS into 6 clusters, two of them were homogenous, DS into 8 clusters, all of them heterogeneous, DNS into 4 clusters, all of them heterogenous. The distribution of subjects from different populations in clusters is presented in Figure 4. Solution was discarded as subjects were not classified according to clinical characteristics.


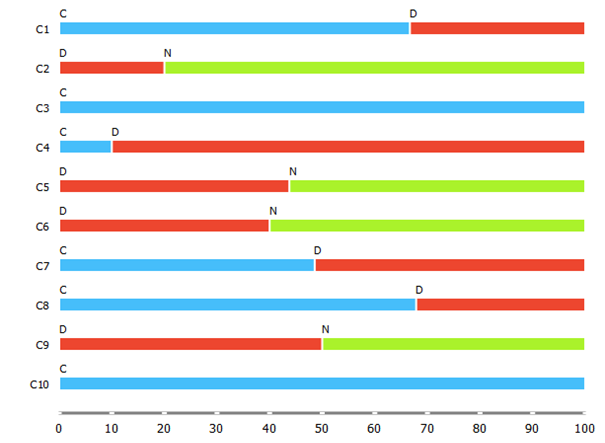


Figure 4. Distributions of percentage of CS, DS and DNS in clusters in Solution 4.

In the Solution 5 input data were all the EMG parameters acquired during stair negotiation only. Six clusters were formed, 5 of them were homogenous. All DS were classified into a single cluster. CS were classified into 2 clusters – a homogenous C2 with 22% of CS and C6 with 78% of CS and 6% of DNS. DNS were classified into a mixed cluster with CS and 3 other homogenous clusters. The distribution of subjects from different populations in clusters is presented in Figure 4. Solution was discarded due to the presence of a mixed cluster with CS and DNS.


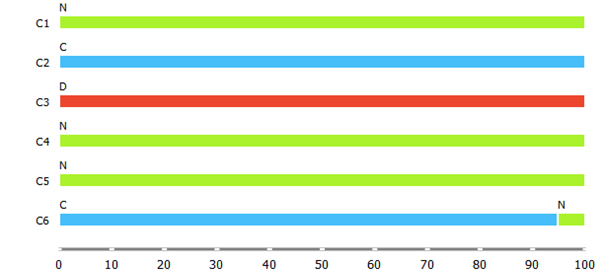


Figure 5. Distributions of percentage of CS, DS and DNS in clusters in Solution 5.

*Table 1. Temporal parameters of gait in analysed subjects, presented as mean and standard deviation of all trials per subject.*

| **Subject** |  | **Duration of gait cycle gait [sec]** | **Duration of stance phase [sec]** | **Duration of swing phase [sec]** | **Duration of stance phase [%]** | **Duration of swing phase [%]** |
| --- | --- | --- | --- | --- | --- | --- |
| **DNS 1** | MEAN | 1.32 | 0.81 | 0.50 | 61.76 | 38.24 |
|  | ST DEV | 0.10 | 0.07 | 0.03 | 1.21 | 1.21 |
| **DNS 2** | MEAN | 1.23 | 0.74 | 0.50 | 59.65 | 40.35 |
|  | ST DEV | 0.04 | 0.04 | 0.01 | 1.19 | 1.19 |
| **DNS 3** | MEAN | 1.27 | 0.76 | 0.51 | 60.17 | 39.83 |
|  | ST DEV | 0.03 | 0.02 | 0.02 | 0.96 | 0.96 |
| **DNS 4** | MEAN | 1.12 | 0.71 | 0.41 | 63.35 | 36.65 |
|  | ST DEV | 0.03 | 0.02 | 0.02 | 0.98 | 0.98 |
| **DNS 5** | MEAN | 1.00 | 0.60 | 0.41 | 59.57 | 40.43 |
|  | ST DEV | 0.02 | 0.01 | 0.02 | 1.14 | 1.14 |
| **DNS 6** | MEAN | 1.00 | 0.61 | 0.39 | 60.76 | 39.24 |
|  | ST DEV | 0.07 | 0.07 | 0.01 | 2.56 | 2.56 |
| **DNS 7** | MEAN | 0.94 | 0.56 | 0.39 | 59.10 | 40.90 |
|  | ST DEV | 0.03 | 0.03 | 0.01 | 1.06 | 1.06 |
| **DNS 8** | MEAN | 1.09 | 0.69 | 0.39 | 63.96 | 36.04 |
|  | ST DEV | 0.07 | 0.05 | 0.03 | 1.00 | 1.00 |
| **DNS 9** | MEAN | 0.97 | 0.58 | 0.39 | 59.98 | 40.02 |
|  | ST DEV | 0.02 | 0.03 | 0.02 | 1.69 | 1.69 |
| **DS 1** | MEAN | 0.92 | 0.53 | 0.40 | 57.07 | 42.93 |
|  | ST DEV | 0.03 | 0.03 | 0.03 | 3.06 | 3.06 |
| **DS 2** | MEAN | 1.36 | 0.93 | 0.43 | 68.24 | 31.76 |
|  | ST DEV | 0.09 | 0.10 | 0.02 | 3.34 | 3.34 |
| **DS 3** | MEAN | 1.08 | 0.65 | 0.43 | 60.27 | 39.73 |
|  | ST DEV | 0.02 | 0.02 | 0.01 | 1.28 | 1.28 |
| **DS 4** | MEAN | 0.88 | 0.51 | 0.37 | 57.71 | 42.29 |
|  | ST DEV | 0.02 | 0.02 | 0.02 | 1.46 | 1.46 |
| **DS 5** | MEAN | 1.02 | 0.59 | 0.43 | 57.77 | 42.23 |
|  | ST DEV | 0.01 | 0.02 | 0.02 | 1.90 | 1.90 |
| **DS 6** | MEAN | 1.15 | 0.68 | 0.47 | 59.30 | 40.70 |
|  | ST DEV | 0.02 | 0.02 | 0.03 | 1.90 | 1.90 |
| **DS 7** | MEAN | 1.11 | 0.66 | 0.45 | 59.47 | 40.53 |
|  | ST DEV | 0.11 | 0.09 | 0.03 | 2.17 | 2.17 |
| **DS 8** | MEAN | N/A | N/A | N/A | N/A | N/A |
|  | ST DEV | N/A | N/A | N/A | N/A | N/A |
| **DS 9** | MEAN | 1.10 | 0.65 | 0.45 | 59.24 | 40.76 |
|  | ST DEV | 0.03 | 0.02 | 0.01 | 1.09 | 1.09 |
| **CS 1** | MEAN | 1.04 | 0.63 | 0.41 | 60.63 | 39.37 |
|  | ST DEV | 0.09 | 0.07 | 0.02 | 2.17 | 2.17 |
| **CS 2** | MEAN | 0.97 | 0.58 | 0.39 | 59.75 | 40.25 |
|  | ST DEV | 0.03 | 0.02 | 0.02 | 0.96 | 0.96 |
| **CS 3** | MEAN | 0.95 | 0.57 | 0.38 | 59.91 | 40.09 |
|  | ST DEV | 0.04 | 0.03 | 0.04 | 2.89 | 2.89 |
| **CS 4** | MEAN | 1.12 | 0.64 | 0.48 | 56.89 | 43.11 |
|  | ST DEV | 0.04 | 0.05 | 0.04 | 3.39 | 3.39 |
| **CS 5** | MEAN | 1.15 | 0.69 | 0.46 | 60.13 | 39.87 |
|  | ST DEV | 0.03 | 0.03 | 0.01 | 1.39 | 1.39 |
| **CS 6** | MEAN | 1.11 | 0.66 | 0.45 | 59.79 | 40.21 |
|  | ST DEV | 0.03 | 0.03 | 0.03 | 2.42 | 2.42 |
| **CS 7** | MEAN | 1.07 | 0.63 | 0.44 | 58.86 | 41.14 |
|  | ST DEV | 0.03 | 0.01 | 0.03 | 1.27 | 1.27 |
| **CS 8** | MEAN | 1.18 | 0.72 | 0.46 | 60.94 | 39.06 |
|  | ST DEV | 0.04 | 0.03 | 0.02 | 0.86 | 0.86 |
| **CS 9** | MEAN | 1.03 | 0.60 | 0.43 | 58.07 | 41.93 |
|  | ST DEV | 0.03 | 0.02 | 0.02 | 1.41 | 1.41 |
| **CS 10** | MEAN | 1.11 | 0.66 | 0.44 | 59.75 | 40.25 |
|  | ST DEV | 0.06 | 0.04 | 0.02 | 0.81 | 0.81 |

*Table 2. Normalized value of peak of the envelope and its position within cycle of gait in analysed subjects, presented as mean and standard deviation of all trials per subject.*

| **Subject** |  | **RF PoE Gait [% of mean value]** | **RF PPoE [% of gait cycle]** | **TA PoE [% of mean value]** | **TA PPoE [of gait cycle]** | **PL PoE [% of mean value]** | **PL PPoE [% of gait cycle]** | **GL PoE [% of mean value]** | **GL PPoE [% of gait cycle]** | **MG PoE [% of mean value]** | **MG PPoE [% of gait cycle]** | **EDC PoE [% of mean value]** | **EDC PPoE  [% of gait cycle]** |
| --- | --- | --- | --- | --- | --- | --- | --- | --- | --- | --- | --- | --- | --- |
| **DNS 1** | MEAN | 262.08 | 6.75 | 193.52 | 5.69 | 245.93 | 37.74 | 415.77 | 40.97 | 441.50 | 5.29 | N/A | N/A |
|  | ST DEV | 45.47 | 4.12 | 0.00 | 0.00 | 30.36 | 6.25 | 141.34 | 0.53 | 144.93 | 6.40 | N/A | N/A |
| **DNS 2** | MEAN | N/A | N/A | 237.79 | 53.10 | N/A | N/A | N/A | N/A | N/A | N/A | N/A | N/A |
|  | ST DEV | N/A | N/A | 38.26 | 24.78 | N/A | N/A | N/A | N/A | N/A | N/A | N/A | N/A |
| **DNS 3** | MEAN | 278.58 | 10.09 | 259.52 | 48.12 | 318.99 | 31.75 | N/A | N/A | 399.51 | 2.68 | N/A | N/A |
|  | ST DEV | 50.92 | 14.44 | 50.42 | 27.97 | 51.00 | 6.81 | N/A | N/A | 52.80 | 1.23 | N/A | N/A |
| **DNS 4** | MEAN | 272.47 | 7.43 | 233.98 | 37.84 | 338.30 | 43.44 | 391.94 | 44.84 | 233.71 | 12.19 | 244.93 | 17.26 |
|  | ST DEV | 43.90 | 1.61 | 23.48 | 32.01 | 50.69 | 3.36 | 20.39 | 2.73 | 5.95 | 0.23 | 46.52 | 23.83 |
| **DNS 5** | MEAN | 350.45 | 4.33 | 282.89 | 73.49 | 292.68 | 38.19 | 254.79 | 44.24 | N/A | N/A | 190.19 | 86.45 |
|  | ST DEV | 20.56 | 1.34 | 63.93 | 34.46 | 40.82 | 3.54 | 80.48 | 12.47 | N/A | N/A | 39.93 | 11.94 |
| **DNS 6** | MEAN | N/A | N/A | 217.09 | 59.67 | 257.06 | 39.60 | 463.75 | 42.20 | 489.13 | 6.49 | N/A | N/A |
|  | ST DEV | N/A | N/A | 19.78 | 3.77 | 28.37 | 4.24 | 77.76 | 2.20 | 23.43 | 5.21 | N/A | N/A |
| **DNS 7** | MEAN | N/A | N/A | 197.46 | 46.50 | 400.56 | 31.86 | 207.72 | 34.91 | N/A | N/A | N/A | N/A |
|  | ST DEV | N/A | N/A | 28.96 | 29.85 | 105.84 | 8.16 | 31.29 | 6.59 | N/A | N/A | N/A | N/A |
| **DNS 8** | MEAN | 225.64 | 3.20 | 243.49 | 54.13 | 235.41 | 27.56 | 281.81 | 43.12 | 259.30 | 11.58 | 271.87 | 69.68 |
|  | ST DEV | 8.55 | 0.60 | 28.79 | 42.53 | 32.60 | 17.97 | 61.97 | 4.15 | 40.42 | 8.15 | 36.67 | 0.93 |
| **DNS 9** | MEAN | N/A | N/A | 256.67 | 43.03 | N/A | N/A | 264.02 | 22.30 | N/A | N/A | 251.63 | 45.25 |
|  | ST DEV | N/A | N/A | 37.67 | 35.43 | N/A | N/A | 39.74 | 9.12 | N/A | N/A | 21.70 | 5.46 |
| **DS 1** | MEAN | 280.01 | 3.44 | 243.01 | 34.94 | 266.12 | 37.19 | 259.37 | 33.54 | N/A | N/A | N/A | N/A |
|  | ST DEV | 32.31 | 1.46 | 9.65 | 33.04 | 36.65 | 2.84 | 40.12 | 2.89 | N/A | N/A | N/A | N/A |
| **DS 2** | MEAN | 306.55 | 10.08 | 233.04 | 50.68 | N/A | N/A | 377.41 | 43.71 | 363.52 | 69.78 | 206.94 | 85.18 |
|  | ST DEV | 36.11 | 2.07 | 51.78 | 29.93 | N/A | N/A | 62.94 | 2.00 | 0.00 | 0.00 | 0.00 | 0.00 |
| **DS 3** | MEAN | 326.53 | 26.34 | 231.53 | 40.27 | 333.37 | 34.51 | 266.77 | 27.14 | 383.28 | 2.95 | 281.32 | 58.14 |
|  | ST DEV | 48.63 | 18.46 | 37.61 | 32.55 | 59.40 | 13.99 | 65.43 | 14.65 | 38.94 | 1.11 | 33.47 | 25.17 |
| **DS 4** | MEAN | N/A | N/A | 322.33 | 62.27 | 292.59 | 34.27 | 343.17 | 34.57 | N/A | N/A | 255.52 | 75.99 |
|  | ST DEV | N/A | N/A | 24.39 | 8.08 | 12.44 | 7.82 | 52.12 | 7.88 | N/A | N/A | 0.00 | 0.00 |
| **DS 5** | MEAN | N/A | N/A | 230.95 | 56.50 | 294.34 | 21.56 | 360.81 | 35.19 | 196.34 | 30.19 | N/A | N/A |
|  | ST DEV | N/A | N/A | 0.00 | 0.00 | 70.89 | 15.28 | 31.12 | 2.60 | 0.00 | 0.00 | N/A | N/A |
| **DS 6** | MEAN | 167.82 | 7.61 | 293.85 | 30.78 | 354.92 | 46.54 | 388.71 | 41.52 | N/A | N/A | N/A | N/A |
|  | ST DEV | 0.00 | 0.00 | 33.83 | 24.46 | 32.39 | 0.23 | 43.79 | 4.93 | N/A | N/A | N/A | N/A |
| **DS 7** | MEAN | 299.05 | 9.22 | N/A | N/A | N/A | N/A | 344.92 | 36.17 | N/A | N/A | N/A | N/A |
|  | ST DEV | 30.56 | 2.34 | N/A | N/A | N/A | N/A | 15.90 | 4.70 | N/A | N/A | N/A | N/A |
| **DS 8** | MEAN | N/A | N/A | 326.81 | 40.17 | 239.29 | 38.32 | 348.52 | 47.75 | 469.87 | 5.89 | 259.16 | 59.22 |
|  | ST DEV | N/A | N/A | 26.90 | 29.85 | 0.00 | 0.00 | 20.54 | 1.45 | 28.07 | 2.59 | 46.64 | 42.13 |
| **DS 9** | MEAN | N/A | N/A | 282.80 | 35.16 | 298.81 | 36.40 | 269.69 | 38.75 | 412.01 | 4.06 | N/A | N/A |
|  | ST DEV | N/A | N/A | 42.71 | 42.50 | 36.43 | 7.86 | 32.42 | 4.90 | 14.01 | 0.52 | N/A | N/A |
| **CS 1** | MEAN | 198.65 | 10.23 | 271.65 | 32.55 | 167.23 | 12.10 | 284.11 | 49.63 | 294.02 | 9.70 | N/A | N/A |
|  | ST DEV | 37.62 | 5.34 | 62.75 | 43.58 | 0.00 | 0.00 | 49.49 | 9.89 | 11.37 | 4.87 | N/A | N/A |
| **CS 2** | MEAN | 204.01 | 41.36 | 239.77 | 39.09 | 159.19 | 42.80 | 247.09 | 53.17 | 238.80 | 31.84 | N/A | N/A |
|  | ST DEV | 32.83 | 13.74 | 52.54 | 23.82 | 0.00 | 0.00 | 16.30 | 17.61 | 28.54 | 12.29 | N/A | N/A |
| **CS 3** | MEAN | 276.94 | 33.05 | 265.40 | 58.63 | N/A | N/A | 244.04 | 43.43 | 289.91 | 2.99 | N/A | N/A |
|  | ST DEV | 31.82 | 18.93 | 27.54 | 24.86 | N/A | N/A | 36.06 | 2.30 | 22.36 | 0.80 | N/A | N/A |
| **CS 4** | MEAN | 320.66 | 36.47 | 252.23 | 45.42 | 236.85 | 29.42 | 262.18 | 46.32 | 341.39 | 22.24 | N/A | N/A |
|  | ST DEV | 64.49 | 17.49 | 21.55 | 31.25 | 0.00 | 0.00 | 31.70 | 13.90 | 0.00 | 0.00 | N/A | N/A |
| **CS 5** | MEAN | 301.87 | 9.00 | 281.35 | 42.84 | N/A | N/A | 313.88 | 43.17 | 289.95 | 20.43 | N/A | N/A |
|  | ST DEV | 25.00 | 5.50 | 25.86 | 29.71 | N/A | N/A | 79.09 | 3.46 | 73.40 | 34.49 | N/A | N/A |
| **CS 6** | MEAN | 312.45 | 6.09 | 268.97 | 74.67 | N/A | N/A | 309.57 | 29.32 | 440.44 | 4.95 | 241.76 | 67.10 |
|  | ST DEV | 0.00 | 0.00 | 45.76 | 32.29 | N/A | N/A | 59.11 | 13.78 | 30.54 | 0.64 | 26.44 | 2.26 |
| **CS 7** | MEAN | N/A | N/A | 252.87 | 1.55 | 231.81 | 47.16 | 374.02 | 39.71 | 326.51 | 15.79 | 286.07 | 68.86 |
|  | ST DEV | N/A | N/A | 17.96 | 1.08 | 28.33 | 7.85 | 41.63 | 7.43 | 70.64 | 14.58 | 0.00 | 0.00 |
| **CS 8** | MEAN | 270.25 | 1.74 | 276.34 | 70.14 | 254.32 | 38.54 | 231.25 | 2.50 | 434.92 | 26.87 | N/A | N/A |
|  | ST DEV | 0.00 | 0.00 | 9.79 | 0.33 | 32.85 | 6.22 | 0.00 | 0.00 | 0.00 | 0.00 | N/A | N/A |
| **CS 9** | MEAN | 187.20 | 64.84 | 246.00 | 57.04 | 257.51 | 28.43 | 306.30 | 43.90 | 334.42 | 11.56 | N/A | N/A |
|  | ST DEV | 83.19 | 1.63 | 30.04 | 32.93 | 31.26 | 15.61 | 66.12 | 1.86 | 41.69 | 9.93 | N/A | N/A |
| **CS 10** | MEAN | 257.30 | 68.04 | 246.66 | 2.36 | N/A | N/A | 406.35 | 39.75 | 286.72 | 97.91 | N/A | N/A |
|  | ST DEV | 0.00 | 0.00 | 0.00 | 0.00 | N/A | N/A | 35.03 | 1.37 | 0.00 | 0.00 | N/A | N/A |

*Table 3. Onset and offset of muscle activity as a percentage of cycle of gait in analysed subjects, presented as mean and standard deviation of all trials per subject.*

| **Subject** |  | **RF onset Gait [%]** | **RF offset Gait [%]** | **TA onset Gait [%]** | **TA offset Gait [%]** | **PL onset Gait [%]** | **PL offset Gait [%]** | **GM onset Gait [%]** | **GM offset Gait [%]** | **GAL onset Gait [%]** | | **GAL offset Gait [%]** | **EXD onset Gait [%]** | **EXD offset Gait [%]** |
| --- | --- | --- | --- | --- | --- | --- | --- | --- | --- | --- | --- | --- | --- | --- |
| **DNS 1** | MEAN | 0.11 | 0.28 | 0.14 | 0.38 | 0.19 | 0.35 | 0.16 | 0.28 | 0.32 | 0.36 | | 0.11 | 0.46 |
|  | ST DEV | 0.21 | 0.19 | 0.09 | 0.30 | 0.18 | 0.17 | 0.23 | 0.25 | 0.15 | 0.16 | | 0.05 | 0.25 |
| **DNS 2** | MEAN | 0.20 | 0.33 | 0.44 | 0.63 | 0.35 | 0.50 | 0.18 | 0.28 | 0.44 | 0.57 | | 0.29 | 0.38 |
|  | ST DEV | 0.24 | 0.24 | 0.30 | 0.22 | 0.15 | 0.23 | 0.16 | 0.17 | 0.23 | 0.26 | | 0.17 | 0.19 |
| **DNS 3** | MEAN | 0.00 | 0.31 | 0.55 | 0.83 | 0.21 | 0.40 | 0.00 | 0.19 | 0.16 | 0.30 | | 0.18 | 0.44 |
|  | ST DEV | 0.00 | 0.16 | 0.03 | 0.12 | 0.12 | 0.09 | 0.00 | 0.04 | 0.20 | 0.18 | | 0.13 | 0.28 |
| **DNS 4** | MEAN | 0.02 | 0.42 | 0.53 | 0.65 | 0.24 | 0.53 | 0.05 | 0.21 | 0.25 | 0.52 | | 0.28 | 0.36 |
|  | ST DEV | 0.03 | 0.18 | 0.27 | 0.28 | 0.12 | 0.10 | 0.06 | 0.15 | 0.18 | 0.08 | | 0.16 | 0.20 |
| **DNS 5** | MEAN | 0.00 | 0.13 | 0.61 | 0.76 | 0.08 | 0.41 | 0.17 | 0.42 | 0.19 | 0.35 | | 0.41 | 0.51 |
|  | ST DEV | 0.00 | 0.02 | 0.02 | 0.01 | 0.09 | 0.11 | 0.24 | 0.27 | 0.15 | 0.14 | | 0.20 | 0.21 |
| **DNS 6** | MEAN | 0.00 | 0.19 | 0.43 | 0.58 | 0.07 | 0.43 | 0.15 | 0.27 | 0.24 | 0.35 | | 0.21 | 0.43 |
|  | ST DEV | 0.00 | 0.09 | 0.16 | 0.23 | 0.07 | 0.11 | 0.34 | 0.30 | 0.15 | 0.19 | | 0.15 | 0.22 |
| **DNS 7** | MEAN | 0.02 | 0.20 | 0.46 | 0.69 | 0.19 | 0.33 | 0.14 | 0.28 | 0.09 | 0.42 | | 0.45 | 0.66 |
|  | ST DEV | 0.04 | 0.11 | 0.13 | 0.23 | 0.10 | 0.09 | 0.32 | 0.32 | 0.11 | 0.07 | | 0.21 | 0.31 |
| **DNS 8** | MEAN | 0.04 | 0.26 | 0.50 | 0.59 | 0.29 | 0.50 | 0.03 | 0.41 | 0.15 | 0.35 | | 0.52 | 0.68 |
|  | ST DEV | 0.06 | 0.08 | 0.22 | 0.23 | 0.15 | 0.09 | 0.06 | 0.07 | 0.18 | 0.14 | | 0.16 | 0.18 |
| **DNS 9** | MEAN | 0.13 | 0.33 | 0.55 | 0.70 | 0.22 | 0.49 | 0.00 | 0.23 | 0.09 | 0.41 | | 0.33 | 0.47 |
|  | ST DEV | 0.26 | 0.26 | 0.22 | 0.20 | 0.11 | 0.14 | 0.00 | 0.07 | 0.03 | 0.12 | | 0.11 | 0.14 |
| **DS 1** | MEAN | 0.40 | 0.46 | 0.45 | 0.59 | 0.33 | 0.56 | 0.34 | 0.46 | 0.27 | 0.48 | | 0.43 | 0.64 |
|  | ST DEV | 0.22 | 0.24 | 0.34 | 0.37 | 0.32 | 0.27 | 0.37 | 0.40 | 0.26 | 0.19 | | 0.31 | 0.26 |
| **DS 2** | MEAN | 0.42 | 0.71 | 0.42 | 0.62 | 0.32 | 0.63 | 0.41 | 0.50 | 0.25 | 0.51 | | 0.43 | 0.61 |
|  | ST DEV | 0.33 | 0.23 | 0.29 | 0.29 | 0.31 | 0.20 | 0.30 | 0.28 | 0.29 | 0.24 | | 0.30 | 0.32 |
| **DS 3** | MEAN | 0.31 | 0.45 | 0.41 | 0.64 | 0.40 | 0.53 | 0.29 | 0.46 | 0.42 | 0.63 | | 0.42 | 0.67 |
|  | ST DEV | 0.19 | 0.21 | 0.32 | 0.40 | 0.30 | 0.27 | 0.35 | 0.33 | 0.36 | 0.22 | | 0.30 | 0.34 |
| **DS 4** | MEAN | 0.32 | 0.41 | 0.50 | 0.67 | 0.28 | 0.53 | 0.39 | 0.55 | 0.10 | 0.44 | | 0.49 | 0.66 |
|  | ST DEV | 0.35 | 0.35 | 0.30 | 0.28 | 0.29 | 0.30 | 0.44 | 0.42 | 0.08 | 0.07 | | 0.29 | 0.28 |
| **DS 5** | MEAN | 0.26 | 0.35 | 0.43 | 0.60 | 0.23 | 0.46 | 0.39 | 0.51 | 0.29 | 0.57 | | 0.44 | 0.60 |
|  | ST DEV | 0.27 | 0.26 | 0.33 | 0.32 | 0.25 | 0.18 | 0.37 | 0.33 | 0.36 | 0.23 | | 0.32 | 0.33 |
| **DS 6** | MEAN | 0.31 | 0.40 | 0.39 | 0.62 | 0.35 | 0.56 | 0.35 | 0.42 | 0.15 | 0.48 | | 0.38 | 0.47 |
|  | ST DEV | 0.38 | 0.32 | 0.33 | 0.29 | 0.33 | 0.27 | 0.38 | 0.36 | 0.12 | 0.15 | | 0.33 | 0.36 |
| **DS 7** | MEAN | 0.43 | 0.50 | 0.33 | 0.62 | 0.22 | 0.41 | 0.46 | 0.53 | 0.05 | 0.42 | | 0.31 | 0.72 |
|  | ST DEV | 0.37 | 0.34 | 0.32 | 0.35 | 0.28 | 0.24 | 0.41 | 0.39 | 0.08 | 0.04 | | 0.30 | 0.34 |
| **DS 8** | MEAN | N/A | N/A | N/A | N/A | N/A | N/A | N/A | N/A | N/A | N/A | | N/A | N/A |
|  | ST DEV | N/A | N/A | N/A | N/A | N/A | N/A | N/A | N/A | N/A | N/A | | N/A | N/A |
| **DS 9** | MEAN | 0.53 | 0.58 | 0.32 | 0.54 | 0.36 | 0.59 | 0.34 | 0.51 | 0.24 | 0.60 | | 0.35 | 0.55 |
|  | ST DEV | 0.40 | 0.40 | 0.28 | 0.40 | 0.39 | 0.25 | 0.33 | 0.26 | 0.28 | 0.15 | | 0.24 | 0.32 |
| **CS 1** | MEAN | 0.30 | 0.44 | 0.40 | 0.61 | 0.25 | 0.46 | 0.56 | 0.76 | N/A | N/A | | N/A | N/A |
|  | ST DEV | 0.42 | 0.40 | 0.27 | 0.26 | 0.25 | 0.29 | 0.26 | 0.34 | N/A | N/A | | N/A | N/A |
| **CS 2** | MEAN | 0.07 | 0.28 | 0.55 | 0.85 | 0.45 | 0.55 | 0.06 | 0.44 | N/A | N/A | | N/A | N/A |
|  | ST DEV | 0.15 | 0.17 | 0.18 | 0.31 | 0.16 | 0.19 | 0.12 | 0.04 | N/A | N/A | | N/A | N/A |
| **CS 3** | MEAN | 0.38 | 0.50 | 0.32 | 0.57 | 0.11 | 0.48 | 0.29 | 0.43 | 0.34 | 0.53 | | 0.40 | 0.64 |
|  | ST DEV | 0.18 | 0.23 | 0.27 | 0.37 | 0.22 | 0.08 | 0.40 | 0.41 | 0.16 | 0.08 | | 0.15 | 0.24 |
| **CS 4** | MEAN | 0.35 | 0.49 | 0.38 | 0.58 | 0.28 | 0.54 | 0.36 | 0.56 | 0.54 | 0.81 | | 0.41 | 0.64 |
|  | ST DEV | 0.23 | 0.24 | 0.21 | 0.20 | 0.21 | 0.15 | 0.20 | 0.26 | 0.20 | 0.20 | | 0.17 | 0.12 |
| **CS 5** | MEAN | 0.04 | 0.23 | 0.57 | 1.00 | 0.25 | 0.47 | 0.01 | 0.38 | 0.23 | 0.35 | | 0.46 | 0.62 |
|  | ST DEV | 0.05 | 0.06 | 0.02 | 0.01 | 0.16 | 0.05 | 0.01 | 0.03 | 0.16 | 0.24 | | 0.16 | 0.17 |
| **CS 6** | MEAN | 0.08 | 0.20 | 0.40 | 0.70 | 0.19 | 0.43 | 0.10 | 0.26 | 0.07 | 0.37 | | 0.49 | 0.63 |
|  | ST DEV | 0.16 | 0.17 | 0.25 | 0.31 | 0.18 | 0.10 | 0.14 | 0.26 | 0.11 | 0.13 | | 0.22 | 0.27 |
| **CS 7** | MEAN | 0.27 | 0.58 | 0.47 | 0.57 | 0.21 | 0.49 | 0.04 | 0.42 | 0.18 | 0.48 | | 0.54 | 0.68 |
|  | ST DEV | 0.23 | 0.16 | 0.22 | 0.26 | 0.16 | 0.07 | 0.04 | 0.09 | 0.16 | 0.07 | | 0.18 | 0.20 |
| **CS 8** | MEAN | 0.11 | 0.45 | 0.54 | 0.90 | 0.22 | 0.52 | 0.03 | 0.38 | 0.27 | 0.67 | | 0.28 | 0.65 |
|  | ST DEV | 0.21 | 0.31 | 0.24 | 0.31 | 0.20 | 0.28 | 0.02 | 0.24 | 0.26 | 0.32 | | 0.14 | 0.16 |
| **CS 9** | MEAN | 0.18 | 0.31 | 0.62 | 0.75 | 0.24 | 0.48 | 0.28 | 0.41 | 0.31 | 0.54 | | 0.46 | 0.67 |
|  | ST DEV | 0.21 | 0.19 | 0.02 | 0.02 | 0.20 | 0.05 | 0.27 | 0.25 | 0.19 | 0.09 | | 0.18 | 0.24 |
| **CS 10** | MEAN | 0.79 | 0.87 | 0.60 | 0.97 | 0.32 | 0.51 | 0.06 | 0.21 | 0.38 | 0.62 | | 0.42 | 0.62 |
|  | ST DEV | 0.12 | 0.10 | 0.03 | 0.05 | 0.26 | 0.35 | 0.05 | 0.15 | 0.22 | 0.17 | | 0.29 | 0.34 |

*Table 4. Duration of muscle activity in analysed subjects, presented as mean and standard deviation of all trials per subject.*

| **Subject** |  | **RF Duration Gait [ms]** | **TA Duration Gait [ms]** | **PL Duration Gait [ms]** | **GM Duration Gait [ms]** | **GAL Duration Gait [ms]** | **EDC Duration Gait [ms]** |
| --- | --- | --- | --- | --- | --- | --- | --- |
| **DNS 1** | MEAN | 302.94 | 274.33 | 256.22 | 341.72 | 93.11 | 519.67 |
|  | ST DEV | 255.91 | 292.14 | 283.07 | 339.89 | 70.50 | 615.09 |
| **DNS 2** | MEAN | 422.33 | 787.33 | 224.28 | 232.56 | 239.50 | 180.28 |
|  | ST DEV | 408.11 | 740.43 | 201.37 | 157.56 | 235.12 | 145.29 |
| **DNS 3** | MEAN | 512.72 | 330.28 | 153.72 | 269.72 | 281.39 | 302.06 |
|  | ST DEV | 269.72 | 247.49 | 171.77 | 141.39 | 219.78 | 312.68 |
| **DNS 4** | MEAN | 364.00 | 194.94 | 261.06 | 194.28 | 272.72 | 187.67 |
|  | ST DEV | 217.90 | 141.68 | 174.25 | 164.13 | 155.44 | 160.15 |
| **DNS 5** | MEAN | 224.78 | 155.44 | 396.06 | 272.39 | 165.39 | 108.67 |
|  | ST DEV | 92.30 | 61.70 | 173.23 | 282.52 | 126.98 | 85.68 |
| **DNS 6** | MEAN | 327.83 | 259.39 | 296.28 | 226.22 | 100.94 | 255.44 |
|  | ST DEV | 231.53 | 189.14 | 213.46 | 410.29 | 77.32 | 203.04 |
| **DNS 7** | MEAN | 217.28 | 281.39 | 198.61 | 175.28 | 246.17 | 207.06 |
|  | ST DEV | 150.91 | 183.06 | 202.49 | 118.85 | 162.81 | 192.39 |
| **DNS 8** | MEAN | 236.22 | 125.67 | 192.39 | 389.61 | 367.50 | 117.94 |
|  | ST DEV | 215.92 | 95.05 | 202.21 | 195.94 | 293.50 | 77.49 |
| **DNS 9** | MEAN | 200.44 | 209.78 | 164.89 | 174.44 | 255.94 | 149.00 |
|  | ST DEV | 169.78 | 160.23 | 168.04 | 105.10 | 138.48 | 79.34 |
| **DS 1** | MEAN | 108.39 | 346.89 | 238.00 | 145.17 | 257.78 | 255.83 |
|  | ST DEV | 179.66 | 283.51 | 211.57 | 55.27 | 100.54 | 249.94 |
| **DS 2** | MEAN | 580.83 | 228.33 | 277.67 | 156.28 | 342.56 | 143.72 |
|  | ST DEV | 540.41 | 195.16 | 307.02 | 151.33 | 254.89 | 117.75 |
| **DS 3** | MEAN | 209.72 | 421.50 | 326.44 | 247.72 | 269.83 | 193.39 |
|  | ST DEV | 381.26 | 243.40 | 603.16 | 277.36 | 146.83 | 224.40 |
| **DS 4** | MEAN | 214.22 | 335.78 | 360.94 | 204.00 | 392.56 | 161.56 |
|  | ST DEV | 155.15 | 312.20 | 193.56 | 119.75 | 97.56 | 147.21 |
| **DS 5** | MEAN | 169.33 | 341.11 | 172.56 | 276.33 | 116.22 | 298.28 |
|  | ST DEV | 62.62 | 291.51 | 177.00 | 163.14 | 91.59 | 257.98 |
| **DS 6** | MEAN | 163.72 | 258.17 | 327.50 | 186.50 | 291.39 | 73.89 |
|  | ST DEV | 89.03 | 231.36 | 245.77 | 199.49 | 127.23 | 61.06 |
| **DS 7** | MEAN | 82.61 | 183.44 | 248.06 | 203.67 | 302.28 | 438.67 |
|  | ST DEV | 57.00 | 145.41 | 189.45 | 207.97 | 122.80 | 373.66 |
| **DS 8** | MEAN | 183.72 | 247.44 | 206.11 | N/A | 240.78 | N/A |
|  | ST DEV | 159.00 | 337.06 | 130.35 | N/A | 197.65 | N/A |
| **DS 9** | MEAN | 177.39 | 254.39 | 321.72 | 254.50 | 188.78 | 296.06 |
|  | ST DEV | 145.71 | 222.31 | 293.82 | 282.73 | 177.79 | 196.98 |
| **CS 1** | MEAN | 244.22 | 273.94 | 228.06 | 620.50 | N/A | N/A |
|  | ST DEV | 157.09 | 334.16 | 185.38 | 410.42 | N/A | N/A |
| **CS 2** | MEAN | 339.89 | 494.61 | 195.83 | 465.11 | N/A | N/A |
|  | ST DEV | 256.76 | 234.43 | 159.81 | 237.47 | N/A | N/A |
| **CS 3** | MEAN | 133.44 | 283.33 | 384.11 | 183.39 | 186.17 | 261.17 |
|  | ST DEV | 103.89 | 206.01 | 105.56 | 172.50 | 193.09 | 204.76 |
| **CS 4** | MEAN | 139.22 | 255.83 | 214.17 | 217.44 | 233.44 | 240.72 |
|  | ST DEV | 86.97 | 179.92 | 176.32 | 310.80 | 152.38 | 264.72 |
| **CS 5** | MEAN | 206.67 | 465.56 | 284.28 | 400.94 | 168.67 | 247.17 |
|  | ST DEV | 114.11 | 104.01 | 207.46 | 168.02 | 195.16 | 165.18 |
| **CS 6** | MEAN | 171.78 | 337.50 | 272.67 | 206.39 | 250.61 | 194.72 |
|  | ST DEV | 99.10 | 189.80 | 208.10 | 155.53 | 221.76 | 148.47 |
| **CS 7** | MEAN | 360.56 | 219.72 | 209.33 | 495.56 | 229.11 | 269.22 |
|  | ST DEV | 268.26 | 183.00 | 174.43 | 143.49 | 159.25 | 180.95 |
| **CS 8** | MEAN | 418.17 | 566.72 | 374.50 | 313.67 | 483.22 | 567.94 |
|  | ST DEV | 426.71 | 362.38 | 647.35 | 230.54 | 426.96 | 417.03 |
| **CS 9** | MEAN | 265.17 | 159.72 | 196.06 | 131.94 | 256.83 | 285.67 |
|  | ST DEV | 328.36 | 102.98 | 166.46 | 106.57 | 203.91 | 223.83 |
| **CS 10** | MEAN | 95.78 | 384.72 | 110.89 | 170.00 | 438.00 | 253.39 |
|  | ST DEV | 49.52 | 252.50 | 137.23 | 108.15 | 349.12 | 272.50 |

*Table 5. Temporal parameters of stair climbing in analysed subjects, presented as mean and standard deviation of all trials per subject.*

| **Subject** |  | **Duration Low Step Up** | **Duration Low Step Down** | **Duration High Step Up** | **Duration High step Down** |
| --- | --- | --- | --- | --- | --- |
| **DNS 1** | MEAN | 0.57 | 0.60 | 0.79 | 0.58 |
|  | ST DEV | 0.04 | 0.11 | 0.12 | 0.03 |
| **DNS 2** | MEAN | 0.68 | 0.54 | 0.58 | 0.54 |
|  | ST DEV | 0.01 | 0.06 | 0.09 | 0.03 |
| **DNS 3** | MEAN | 0.54 | 0.53 | 0.68 | 0.58 |
|  | ST DEV | 0.03 | 0.06 | 0.08 | 0.08 |
| **DNS 4** | MEAN | N/A | N/A | N/A | N/A |
|  | ST DEV | N/A | N/A | N/A | N/A |
| **DNS 5** | MEAN | 0.60 | 0.60 | 0.80 | 0.65 |
|  | ST DEV | 0.00 | 0.00 | 0.00 | 0.00 |
| **DNS 6** | MEAN | 0.61 | 0.44 | 0.66 | 0.46 |
|  | ST DEV | 0.12 | 0.07 | 0.14 | 0.04 |
| **DNS 7** | MEAN | N/A | N/A | N/A | N/A |
|  | ST DEV | N/A | N/A | N/A | N/A |
| **DNS 8** | MEAN | N/A | N/A | N/A | N/A |
|  | ST DEV | N/A | N/A | N/A | N/A |
| **DNS 9** | MEAN | 0.69 | 0.67 | 0.73 | 0.70 |
|  | ST DEV | 0.02 | 0.05 | 0.04 | 0.04 |
| **DS 1** | MEAN | 0.57 | 0.49 | 0.66 | 0.52 |
|  | ST DEV | 0.06 | 0.03 | 0.03 | 0.05 |
| **DS 2** | MEAN | 0.68 | 0.60 | 0.74 | 0.72 |
|  | ST DEV | 0.06 | 0.06 | 0.10 | 0.09 |
| **DS 3** | MEAN | 0.50 | 0.63 | 0.60 | 0.66 |
|  | ST DEV | 0.06 | 0.13 | 0.00 | 0.09 |
| **DS 4** | MEAN | 0.62 | 0.56 | 0.53 | 0.43 |
|  | ST DEV | 0.06 | 0.06 | 0.02 | 0.05 |
| **DS 5** | MEAN | 0.46 | 0.42 | 0.52 | 0.53 |
|  | ST DEV | 0.05 | 0.02 | 0.03 | 0.03 |
| **DS 6** | MEAN | 0.53 | 0.58 | 0.52 | 0.63 |
|  | ST DEV | 0.07 | 0.02 | 0.02 | 0.08 |
| **DS 7** | MEAN | 0.40 | 0.48 | 0.49 | 0.50 |
|  | ST DEV | 0.04 | 0.06 | 0.05 | 0.05 |
| **DS 8** | MEAN | 0.68 | 0.60 | 0.63 | 0.60 |
|  | ST DEV | 0.01 | 0.05 | 0.03 | 0.02 |
| **DS 9** | MEAN | 0.56 | 0.57 | 0.58 | 0.49 |
|  | ST DEV | 0.06 | 0.00 | 0.00 | 0.01 |
| **CS 1** | MEAN | 0.60 | 0.66 | 0.78 | 0.77 |
|  | ST DEV | 0.08 | 0.12 | 0.15 | 0.09 |
| **CS 2** | MEAN | 0.80 | 0.62 | 0.78 | 0.68 |
|  | ST DEV | 0.05 | 0.01 | 0.09 | 0.06 |
| **CS 3** | MEAN | 0.66 | 0.72 | 0.89 | 0.94 |
|  | ST DEV | 0.05 | 0.02 | 0.17 | 0.24 |
| **CS 4** | MEAN | 0.60 | 0.61 | 0.59 | 0.58 |
|  | ST DEV | 0.02 | 0.14 | 0.02 | 0.01 |
| **CS 5** | MEAN | 0.60 | 0.63 | 0.78 | 0.66 |
|  | ST DEV | 0.03 | 0.07 | 0.17 | 0.07 |
| **CS 6** | MEAN | 0.55 | 0.68 | 0.61 | 0.70 |
|  | ST DEV | 0.02 | 0.06 | 0.11 | 0.07 |
| **CS 7** | MEAN | 0.67 | 0.49 | 0.59 | 0.52 |
|  | ST DEV | 0.14 | 0.07 | 0.01 | 0.05 |
| **CS 8** | MEAN | 0.55 | 0.47 | 0.63 | 0.47 |
|  | ST DEV | 0.05 | 0.04 | 0.05 | 0.12 |
| **CS 9** | MEAN | 0.62 | 0.68 | 0.61 | 0.76 |
|  | ST DEV | 0.03 | 0.04 | 0.06 | 0.05 |
| **CS 10** | MEAN | N/A | N/A | N/A | N/A |
|  | ST DEV | N/A | N/A | N/A | N/A |

*Table 6. Duration of muscle activity during stair ascent in analysed subjects, presented as mean and standard deviation of all trials per subject.*

| **Subject** |  | **RF Duration Step [ms]** | **TA Duration Step [ms]** | **PL Duration Step [ms]** | **GM Duration Step [ms]** | **GAL Duration Step [ms]** | **EDC Duration Step [ms]** |
| --- | --- | --- | --- | --- | --- | --- | --- |
| **DNS 1** | MEAN | 484.44 | 764.56 | 569.67 | 899.67 | 152.83 | 247.50 |
|  | ST DEV | 791.80 | 894.82 | 538.27 | 953.85 | 164.71 | 318.99 |
| **DNS 2** | MEAN | 482.11 | 451.67 | 517.89 | 407.44 | 329.72 | 868.39 |
|  | ST DEV | 536.61 | 387.16 | 589.34 | 515.98 | 367.62 | 808.56 |
| **DNS 3** | MEAN | 618.06 | 234.72 | 260.44 | 471.17 | 230.33 | 330.89 |
|  | ST DEV | 582.23 | 213.95 | 174.62 | 287.19 | 160.69 | 349.91 |
| **DNS 4** | MEAN | N/A | N/A | N/A | N/A | N/A | N/A |
|  | ST DEV | N/A | N/A | N/A | N/A | N/A | N/A |
| **DNS 5** | MEAN | N/A | 378.50 | 219.67 | N/A | 128.00 | 228.00 |
|  | ST DEV | N/A | 35.50 | 145.27 | N/A | 29.00 | 82.57 |
| **DNS 6** | MEAN | 499.56 | 311.83 | 385.00 | 285.89 | 271.17 | 475.56 |
|  | ST DEV | 436.37 | 350.12 | 303.58 | 295.32 | 276.17 | 523.66 |
| **DNS 7** | MEAN | N/A | N/A | N/A | N/A | N/A | N/A |
|  | ST DEV | N/A | N/A | N/A | N/A | N/A | N/A |
| **DNS 8** | MEAN | N/A | N/A | N/A | N/A | N/A | N/A |
|  | ST DEV | N/A | N/A | N/A | N/A | N/A | N/A |
| **DNS 9** | MEAN | 489.39 | 374.72 | 366.78 | 272.11 | 199.33 | 238.28 |
|  | ST DEV | 855.91 | 495.82 | 324.31 | 283.92 | 144.66 | 216.76 |
| **DS 1** | MEAN | 514.72 | 516.94 | 411.22 | 3364.89 | 410.83 | 996.67 |
|  | ST DEV | 907.46 | 335.33 | 272.68 | 388.43 | 203.82 | 415.21 |
| **DS 2** | MEAN | 1485.89 | 292.39 | 190.89 | 442.00 | 284.83 | 356.61 |
|  | ST DEV | 860.93 | 240.82 | 141.58 | 375.78 | 140.52 | 418.64 |
| **DS 3** | MEAN | 356.00 | 277.44 | 157.28 | 290.22 | 188.17 | 266.33 |
|  | ST DEV | 540.32 | 177.17 | 133.52 | 497.52 | 159.76 | 185.18 |
| **DS 4** | MEAN | 167.39 | 390.28 | 134.39 | 447.94 | 417.33 | 328.67 |
|  | ST DEV | 120.49 | 340.09 | 135.55 | 694.96 | 304.28 | 317.89 |
| **DS 5** | MEAN | 194.83 | 252.39 | 241.72 | 242.56 | 143.78 | 84.78 |
|  | ST DEV | 166.71 | 224.37 | 268.17 | 272.64 | 150.12 | 65.93 |
| **DS 6** | MEAN | 133.67 | 234.89 | 264.00 | 199.50 | 188.11 | 115.50 |
|  | ST DEV | 66.20 | 147.93 | 263.33 | 373.07 | 136.94 | 90.08 |
| **DS 7** | MEAN | 135.56 | 696.78 | 209.72 | 172.00 | 294.72 | 369.59 |
|  | ST DEV | 30.70 | 480.65 | 184.17 | 0.00 | 263.87 | 252.07 |
| **DS 8** | MEAN | 366.17 | 441.56 | 316.56 | 387.28 | 300.94 | 416.56 |
|  | ST DEV | 416.88 | 295.81 | 306.40 | 647.01 | 224.41 | 333.02 |
| **DS 9** | MEAN | 700.56 | 450.44 | 169.61 | 1399.72 | 112.50 | 753.94 |
|  | ST DEV | 1175.53 | 308.22 | 114.19 | 987.91 | 96.01 | 104.49 |
| **CS 1** | MEAN | 406.22 | 421.22 | 396.67 | 497.78 | 225.17 | 461.83 |
|  | ST DEV | 291.05 | 421.94 | 269.64 | 318.11 | 337.42 | 445.99 |
| **CS 2** | MEAN | 565.89 | 398.94 | 327.50 | 712.56 | 454.78 | 793.33 |
|  | ST DEV | 408.01 | 207.48 | 316.49 | 367.20 | 410.45 | 973.78 |
| **CS 3** | MEAN | 770.06 | 683.44 | 348.17 | 524.28 | 498.83 | 740.33 |
|  | ST DEV | 832.49 | 637.72 | 331.09 | 663.06 | 345.19 | 785.30 |
| **CS 4** | MEAN | 357.17 | 313.67 | 272.50 | 314.61 | 362.56 | 457.17 |
|  | ST DEV | 334.71 | 196.00 | 204.03 | 282.86 | 327.24 | 454.13 |
| **CS 5** | MEAN | 461.17 | 326.50 | 278.44 | 609.50 | 427.39 | 396.39 |
|  | ST DEV | 407.35 | 290.42 | 237.32 | 379.11 | 380.21 | 374.69 |
| **CS 6** | MEAN | 302.89 | 317.89 | 442.22 | 350.56 | 140.89 | 417.28 |
|  | ST DEV | 335.14 | 299.65 | 366.03 | 307.36 | 165.76 | 212.44 |
| **CS 7** | MEAN | 518.06 | 197.83 | 354.00 | 326.67 | 264.67 | 225.89 |
|  | ST DEV | 437.62 | 185.85 | 278.22 | 228.61 | 252.26 | 169.45 |
| **CS 8** | MEAN | 433.22 | 536.94 | 293.78 | 356.28 | 315.67 | 164.17 |
|  | ST DEV | 371.19 | 623.15 | 209.88 | 263.38 | 306.67 | 110.41 |
| **CS 9** | MEAN | 330.22 | 232.67 | 362.72 | 393.89 | 226.00 | 247.22 |
|  | ST DEV | 424.20 | 213.15 | 334.03 | 279.82 | 191.49 | 201.98 |
| **CS 10** | MEAN | 240.11 | 312.94 | 168.22 | 468.89 | 307.50 | 237.94 |
|  | ST DEV | 223.53 | 270.37 | 162.53 | 323.47 | 304.09 | 198.80 |

*Table 7. Onset and offset of muscle activity during stair ascent as a percentage of stair ascent cycle in analysed subjects, presented as mean and standard deviation of all trials per subject.*

| **Subject** |  | PL Onset Stair Ascent | PL offset Stair Ascent | TA onset Stair Ascent | TA offset Stair Ascent | GAL onset Stair Ascent | GAL offset Stair Ascent | EXD onset Stair Ascent | EXD offset Stair Ascent | GM onset Stair Ascent | GM offset Stair Ascent | RF onset Stair Ascent | RF offset Stair Ascent |
| --- | --- | --- | --- | --- | --- | --- | --- | --- | --- | --- | --- | --- | --- |
| **DNS 1** | MEAN | 0.20 | 0.25 | 0.05 | 0.15 | 0.05 | 0.08 | 0.03 | 0.05 | 0.17 | 0.23 | 0.10 | 0.17 |
|  | ST DEV | 0.06 | 0.11 | 0.04 | 0.15 | 0.07 | 0.06 | 0.03 | 0.04 | 0.12 | 0.18 | 0.11 | 0.16 |
| **DNS 2** | MEAN | 0.12 | 0.13 | 0.08 | 0.13 | 0.20 | 0.26 | 0.06 | 0.11 | 0.20 | 0.29 | 0.18 | 0.23 |
|  | ST DEV | 0.08 | 0.08 | 0.06 | 0.08 | 0.09 | 0.09 | 0.05 | 0.07 | 0.13 | 0.20 | 0.08 | 0.13 |
| **DNS 3** | MEAN | 0.02 | 0.06 | 0.08 | 0.09 | 0.16 | 0.23 | 0.07 | 0.18 | 0.01 | 0.13 | 0.00 | 0.18 |
|  | ST DEV | 0.02 | 0.04 | 0.11 | 0.11 | 0.17 | 0.15 | 0.12 | 0.12 | 0.01 | 0.02 | 0.00 | 0.09 |
| **DNS 4** | MEAN | N/A | N/A | N/A | N/A | N/A | N/A | N/A | N/A | N/A | N/A | N/A | N/A |
|  | ST DEV | N/A | N/A | N/A | N/A | N/A | N/A | N/A | N/A | N/A | N/A | N/A | N/A |
| **DNS 5** | MEAN | 0.32 | 0.60 | 0.34 | 0.78 | 0.28 | 0.47 | 0.40 | 0.73 | N/A | N/A | N/A | N/A |
|  | ST DEV | 0.28 | 0.32 | 0.34 | 0.23 | 0.28 | 0.34 | 0.29 | 0.22 | N/A | N/A | N/A | N/A |
| **DNS 6** | MEAN | 0.00 | 0.07 | 0.00 | 0.07 | 0.03 | 0.06 | 0.01 | 0.09 | 0.01 | 0.08 | 0.01 | 0.11 |
|  | ST DEV | 0.00 | 0.03 | 0.01 | 0.04 | 0.03 | 0.04 | 0.01 | 0.06 | 0.01 | 0.03 | 0.02 | 0.02 |
| **DNS 7** | MEAN | N/A | N/A | N/A | N/A | N/A | N/A | N/A | N/A | N/A | N/A | N/A | N/A |
|  | ST DEV | N/A | N/A | N/A | N/A | N/A | N/A | N/A | N/A | N/A | N/A | N/A | N/A |
| **DNS 8** | MEAN | N/A | N/A | N/A | N/A | N/A | N/A | N/A | N/A | N/A | N/A | N/A | N/A |
|  | ST DEV | N/A | N/A | N/A | N/A | N/A | N/A | N/A | N/A | N/A | N/A | N/A | N/A |
| **DNS 9** | MEAN | 0.02 | 0.11 | 0.12 | 0.20 | 0.12 | 0.15 | 0.15 | 0.20 | 0.10 | 0.18 | 0.04 | 0.09 |
|  | ST DEV | 0.02 | 0.07 | 0.10 | 0.09 | 0.04 | 0.03 | 0.16 | 0.15 | 0.09 | 0.05 | 0.06 | 0.05 |
| **DS 1** | MEAN | 0.15 | 0.68 | 0.04 | 0.39 | 0.01 | 0.20 | 0.00 | 0.15 | 0.01 | 0.12 | 0.24 | 0.41 |
|  | ST DEV | 0.10 | 0.34 | 0.07 | 0.40 | 0.01 | 0.05 | 0.00 | 0.12 | 0.01 | 0.05 | 0.33 | 0.42 |
| **DS 2** | MEAN | 0.03 | 0.14 | 0.08 | 0.39 | 0.02 | 0.09 | 0.06 | 0.49 | 0.16 | 0.21 | 0.13 | 0.53 |
|  | ST DEV | 0.03 | 0.12 | 0.09 | 0.30 | 0.01 | 0.05 | 0.09 | 0.34 | 0.22 | 0.25 | 0.13 | 0.47 |
| **DS 3** | MEAN | 0.00 | 0.21 | 0.07 | 0.66 | 0.26 | 0.48 | 0.03 | 0.30 | 0.17 | 0.24 | 0.23 | 0.36 |
|  | ST DEV | 0.00 | 0.18 | 0.15 | 0.14 | 0.30 | 0.38 | 0.04 | 0.27 | 0.23 | 0.21 | 0.17 | 0.22 |
| **DS 4** | MEAN | 0.11 | 0.19 | 0.03 | 0.33 | 0.19 | 0.47 | 0.00 | 0.33 | 0.42 | 0.84 | 0.17 | 0.30 |
|  | ST DEV | 0.16 | 0.20 | 0.03 | 0.20 | 0.25 | 0.38 | 0.01 | 0.23 | 0.34 | 0.36 | 0.29 | 0.36 |
| **DS 5** | MEAN | 0.07 | 0.25 | 0.09 | 0.36 | 0.18 | 0.30 | 0.08 | 0.26 | 0.03 | 0.10 | 0.09 | 0.31 |
|  | ST DEV | 0.07 | 0.13 | 0.10 | 0.20 | 0.15 | 0.17 | 0.11 | 0.01 | 0.03 | 0.04 | 0.16 | 0.21 |
| **DS 6** | MEAN | 0.00 | 0.05 | 0.00 | 0.10 | 0.15 | 0.29 | 0.00 | 0.08 | 0.06 | 0.14 | 0.29 | 0.37 |
|  | ST DEV | 0.00 | 0.02 | 0.00 | 0.03 | 0.18 | 0.14 | 0.01 | 0.02 | 0.08 | 0.09 | 0.29 | 0.24 |
| **DS 7** | MEAN | 0.15 | 0.21 | 0.08 | 0.15 | 0.18 | 0.31 | 0.17 | 0.28 | 0.18 | 0.22 | 0.14 | 0.20 |
|  | ST DEV | 0.16 | 0.15 | 0.12 | 0.10 | 0.15 | 0.28 | 0.23 | 0.21 | 0.25 | 0.26 | 0.20 | 0.22 |
| **DS 8** | MEAN | 0.00 | 0.05 | 0.00 | 0.03 | 0.01 | 0.04 | 0.01 | 0.05 | 0.06 | 0.11 | 0.11 | 0.17 |
|  | ST DEV | 0.00 | 0.02 | 0.00 | 0.00 | 0.01 | 0.01 | 0.00 | 0.00 | 0.08 | 0.10 | 0.16 | 0.16 |
| **DS 9** | MEAN | 0.24 | 0.39 | 0.00 | 0.03 | 0.21 | 0.27 | 0.08 | 0.26 | 0.00 | 0.08 | 0.08 | 0.37 |
|  | ST DEV | 0.11 | 0.17 | 0.00 | 0.00 | 0.14 | 0.15 | 0.17 | 0.26 | 0.01 | 0.05 | 0.04 | 0.12 |
| **CS 1** | MEAN | 0.01 | 0.10 | 0.03 | 0.10 | 0.10 | 0.15 | 0.03 | 0.10 | 0.00 | 0.12 | 0.00 | 0.09 |
|  | ST DEV | 0.02 | 0.04 | 0.05 | 0.06 | 0.07 | 0.07 | 0.05 | 0.04 | 0.01 | 0.04 | 0.00 | 0.04 |
| **CS 2** | MEAN | 0.05 | 0.16 | 0.13 | 0.20 | 0.03 | 0.17 | 0.03 | 0.14 | 0.02 | 0.21 | 0.01 | 0.18 |
|  | ST DEV | 0.04 | 0.05 | 0.15 | 0.15 | 0.03 | 0.09 | 0.03 | 0.07 | 0.02 | 0.06 | 0.01 | 0.03 |
| **CS 3** | MEAN | 0.03 | 0.08 | 0.09 | 0.15 | 0.18 | 0.24 | 0.00 | 0.12 | 0.00 | 0.10 | 0.00 | 0.13 |
|  | ST DEV | 0.02 | 0.03 | 0.13 | 0.14 | 0.09 | 0.10 | 0.00 | 0.07 | 0.00 | 0.02 | 0.00 | 0.03 |
| **CS 4** | MEAN | 0.19 | 0.26 | 0.04 | 0.12 | 0.19 | 0.25 | 0.04 | 0.11 | 0.00 | 0.09 | 0.00 | 0.10 |
|  | ST DEV | 0.20 | 0.24 | 0.02 | 0.02 | 0.10 | 0.09 | 0.02 | 0.02 | 0.01 | 0.04 | 0.00 | 0.02 |
| **CS 5** | MEAN | 0.06 | 0.12 | 0.11 | 0.16 | 0.06 | 0.19 | 0.03 | 0.11 | 0.03 | 0.21 | 0.03 | 0.19 |
|  | ST DEV | 0.03 | 0.02 | 0.13 | 0.14 | 0.03 | 0.06 | 0.02 | 0.08 | 0.03 | 0.02 | 0.03 | 0.07 |
| **CS 6** | MEAN | 0.01 | 0.12 | 0.01 | 0.07 | 0.11 | 0.13 | 0.01 | 0.09 | 0.03 | 0.16 | 0.02 | 0.15 |
|  | ST DEV | 0.01 | 0.03 | 0.01 | 0.03 | 0.06 | 0.06 | 0.01 | 0.05 | 0.01 | 0.03 | 0.01 | 0.02 |
| **CS 7** | MEAN | 0.01 | 0.09 | 0.10 | 0.14 | 0.06 | 0.14 | 0.04 | 0.09 | 0.01 | 0.08 | 0.00 | 0.14 |
|  | ST DEV | 0.02 | 0.04 | 0.09 | 0.07 | 0.07 | 0.07 | 0.08 | 0.07 | 0.01 | 0.03 | 0.00 | 0.06 |
| **CS 8** | MEAN | 0.04 | 0.06 | 0.03 | 0.09 | 0.08 | 0.12 | 0.10 | 0.12 | 0.04 | 0.11 | 0.01 | 0.11 |
|  | ST DEV | 0.03 | 0.02 | 0.04 | 0.06 | 0.09 | 0.12 | 0.08 | 0.08 | 0.03 | 0.04 | 0.01 | 0.04 |
| **CS 9** | MEAN | 0.02 | 0.10 | 0.05 | 0.10 | 0.15 | 0.20 | 0.03 | 0.08 | 0.01 | 0.12 | 0.00 | 0.10 |
|  | ST DEV | 0.02 | 0.04 | 0.10 | 0.10 | 0.15 | 0.16 | 0.02 | 0.05 | 0.01 | 0.03 | 0.00 | 0.02 |
| **CS 10** | MEAN | 0.05 | 0.10 | 0.25 | 0.29 | 0.06 | 0.17 | 0.15 | 0.20 | 0.05 | 0.23 | 0.01 | 0.10 |
|  | ST DEV | 0.04 | 0.04 | 0.14 | 0.13 | 0.08 | 0.06 | 0.19 | 0.17 | 0.06 | 0.13 | 0.01 | 0.04 |

*Table 8. Normalized value of peak of the envelope and its position within the star ascent cycle in analysed subjects, presented as mean and standard deviation of all trials per subject.*

| **Subject** |  | **RF PoE % UP** | **RF PPoE UP** | **TA PoE % UP** | **TA PPoE UP** | **PL PoE % UP** | **PL PPoE UP** | **GL PoE % UP** | **GL PPoE UP** | **MG PoE % UP** | **MG PPoE UP** | **EDC PoE % UP** | **EDC PPoE UP** |
| --- | --- | --- | --- | --- | --- | --- | --- | --- | --- | --- | --- | --- | --- |
| **DNS 1** | MEAN | 353.57 | 8.13 | 157.91 | 7.71 | 194.55 | 7.23 | 194.30 | 7.06 | 206.24 | 6.77 | 154.73 | 7.10 |
|  | ST DEV | 206.27 | 4.71 | 19.85 | 3.49 | 67.13 | 4.22 | 17.25 | 4.58 | 96.32 | 4.50 | 42.06 | 3.93 |
| **DNS 2** | MEAN | 281.40 | 20.02 | 290.16 | 17.59 | 500.29 | 19.39 | 161.92 | 19.05 | 377.16 | 18.59 | 225.30 | 17.39 |
|  | ST DEV | 158.55 | 2.73 | 140.83 | 3.25 | 47.10 | 3.63 | 27.93 | 3.61 | 291.62 | 3.52 | 44.00 | 3.73 |
| **DNS 3** | MEAN | 414.30 | 21.66 | 379.32 | 21.68 | 458.19 | 22.79 | 323.76 | 19.34 | 162.82 | 20.30 | 170.45 | 21.22 |
|  | ST DEV | 405.99 | 6.08 | 216.51 | 4.91 | 294.43 | 4.33 | 72.09 | 5.43 | 60.34 | 6.35 | 39.75 | 5.28 |
| **DNS 4** | MEAN | N/A | N/A | N/A | N/A | N/A | N/A | N/A | N/A | N/A | N/A | N/A | N/A |
|  | ST DEV | N/A | N/A | N/A | N/A | N/A | N/A | N/A | N/A | N/A | N/A | N/A | N/A |
| **DNS 5** | MEAN | 398.99 | 23.34 | 374.51 | 22.34 | 441.08 | 22.53 | 125.97 | 21.15 | 838.60 | 22.17 | 193.77 | 22.05 |
|  | ST DEV | 34.72 | 1.81 | 163.55 | 3.45 | 0.00 | 1.00 | 21.60 | 3.17 | 257.73 | 0.63 | 89.18 | 3.03 |
| **DNS 6** | MEAN | 140.88 | 19.28 | 244.73 | 16.60 | 331.85 | 17.35 | 166.73 | 16.83 | 524.68 | 18.05 | 139.95 | 17.94 |
|  | ST DEV | 60.42 | 4.48 | 130.42 | 3.88 | 76.97 | 3.59 | 121.10 | 3.89 | 246.16 | 5.18 | 17.23 | 3.35 |
| **DNS 7** | MEAN | N/A | N/A | N/A | N/A | N/A | N/A | N/A | N/A | N/A | N/A | N/A | N/A |
|  | ST DEV | N/A | N/A | N/A | N/A | N/A | N/A | N/A | N/A | N/A | N/A | N/A | N/A |
| **DNS 8** | MEAN | N/A | N/A | N/A | N/A | N/A | N/A | N/A | N/A | N/A | N/A | N/A | N/A |
|  | ST DEV | N/A | N/A | N/A | N/A | N/A | N/A | N/A | N/A | N/A | N/A | N/A | N/A |
| **DNS 9** | MEAN | 124.31 | 21.92 | 438.32 | 20.63 | 428.83 | 21.79 | 113.68 | 21.43 | 274.06 | 21.26 | 235.05 | 21.96 |
|  | ST DEV | 118.21 | 2.98 | 283.05 | 4.38 | 345.44 | 5.48 | 57.01 | 5.99 | 193.66 | 5.20 | 105.44 | 5.80 |
| **DS 1** | MEAN | 210.00 | 25.09 | 167.00 | 25.32 | N/A | 25.01 | 476.79 | 23.74 | 199.78 | 28.53 | 149.82 | 25.61 |
|  | ST DEV | 31.86 | 4.10 | 14.69 | 3.50 | N/A | 2.95 | 296.79 | 2.96 | 20.18 | 3.64 | 16.88 | 3.89 |
| **DS 2** | MEAN | 637.44 | 20.77 | 132.90 | 18.29 | N/A | 19.43 | 706.44 | 17.59 | 87.60 | 19.48 | 127.65 | 18.20 |
|  | ST DEV | 559.30 | 4.26 | 42.61 | 4.43 | N/A | 4.35 | 712.97 | 3.29 | 55.76 | 4.69 | 18.15 | 4.43 |
| **DS 3** | MEAN | 289.25 | 14.65 | 131.34 | 14.03 | N/A | 14.71 | 437.11 | 14.95 | 91.02 | 14.76 | 129.25 | 14.31 |
|  | ST DEV | 168.98 | 2.35 | 20.85 | 3.23 | N/A | 2.87 | 228.00 | 3.36 | 47.83 | 2.77 | 40.93 | 3.48 |
| **DS 4** | MEAN | 228.49 | 25.95 | 218.80 | 20.58 | N/A | 25.99 | 394.69 | 23.79 | 45.53 | 25.95 | 280.98 | 20.30 |
|  | ST DEV | 145.30 | 10.18 | 77.97 | 11.42 | N/A | 10.03 | 413.65 | 10.29 | 16.66 | 10.05 | 73.05 | 11.35 |
| **DS 5** | MEAN | 521.79 | 22.25 | 170.16 | 21.98 | N/A | 21.60 | 493.09 | 21.96 | 90.48 | 23.11 | 147.76 | 21.93 |
|  | ST DEV | 444.12 | 5.40 | 48.91 | 5.81 | N/A | 5.85 | 306.08 | 5.73 | 14.76 | 5.92 | 24.09 | 5.73 |
| **DS 6** | MEAN | 156.53 | 26.70 | 178.49 | 25.39 | N/A | 24.10 | 255.80 | 24.57 | 131.78 | 27.74 | 147.62 | 25.51 |
|  | ST DEV | 36.05 | 6.59 | 8.18 | 6.23 | N/A | 5.07 | 82.88 | 5.09 | 13.32 | 6.98 | 16.22 | 6.04 |
| **DS 7** | MEAN | 178.27 | 22.53 | 266.07 | 22.26 | N/A | 22.83 | 325.34 | 23.52 | 79.27 | 24.17 | 200.52 | 22.84 |
|  | ST DEV | 89.86 | 3.42 | 119.53 | 2.87 | N/A | 2.12 | 109.44 | 2.03 | 16.94 | 3.52 | 53.43 | 3.34 |
| **DS 8** | MEAN | 187.03 | 16.42 | 194.92 | 15.06 | N/A | 14.74 | 464.82 | 15.55 | 89.68 | 14.90 | 162.32 | 15.57 |
|  | ST DEV | 87.80 | 6.05 | 49.62 | 5.97 | N/A | 5.16 | 132.64 | 5.81 | 30.09 | 5.76 | 15.29 | 6.01 |
| **DS 9** | MEAN | 79.34 | 22.86 | 255.05 | 21.83 | N/A | 23.69 | 517.72 | 20.82 | 128.19 | 22.68 | 172.02 | 22.20 |
|  | ST DEV | 51.87 | 4.74 | 40.47 | 5.28 | N/A | 6.88 | 442.89 | 5.15 | 15.27 | 6.30 | 28.60 | 5.36 |
| **CS 1** | MEAN | N/A | N/A | N/A | N/A | N/A | N/A | N/A | N/A | N/A | N/A | N/A | N/A |
|  | ST DEV | N/A | N/A | N/A | N/A | N/A | N/A | N/A | N/A | N/A | N/A | N/A | N/A |
| **CS 2** | MEAN | N/A | N/A | N/A | N/A | N/A | N/A | N/A | N/A | N/A | N/A | N/A | N/A |
|  | ST DEV | N/A | N/A | N/A | N/A | N/A | N/A | N/A | N/A | N/A | N/A | N/A | N/A |
| **CS 3** | MEAN | 357.03 | 17.75 | 145.36 | 15.30 | N/A | 17.26 | 289.11 | 14.19 | 366.20 | 18.10 | 156.02 | 14.82 |
|  | ST DEV | 237.70 | 5.82 | 20.95 | 5.85 | N/A | 5.96 | 183.45 | 4.56 | 153.33 | 5.52 | 12.31 | 5.40 |
| **CS 4** | MEAN | 224.55 | 23.96 | 222.44 | 19.91 | N/A | 24.10 | 27.02 | 21.82 | 131.57 | 23.69 | 224.41 | 21.24 |
|  | ST DEV | 151.00 | 3.68 | 86.53 | 3.66 | N/A | 3.72 | 24.19 | 2.92 | 75.52 | 3.88 | 50.05 | 4.03 |
| **CS 5** | MEAN | 102.30 | 22.66 | 68.93 | 22.27 | N/A | 21.51 | 121.75 | 20.00 | 165.64 | 22.21 | 82.15 | 21.59 |
|  | ST DEV | 54.93 | 4.94 | 9.25 | 3.80 | N/A | 5.48 | 78.12 | 6.15 | 110.22 | 5.27 | 8.77 | 3.21 |
| **CS 6** | MEAN | 270.52 | 22.77 | 60.86 | 22.74 | N/A | 21.75 | 111.33 | 20.83 | 325.02 | 22.49 | 80.31 | 22.65 |
|  | ST DEV | 174.41 | 5.01 | 23.87 | 4.70 | N/A | 5.79 | 77.89 | 3.63 | 210.67 | 5.10 | 22.28 | 5.38 |
| **CS 7** | MEAN | 206.72 | 23.38 | 97.27 | 20.27 | N/A | 21.42 | 281.49 | 22.56 | 130.18 | 21.82 | 122.89 | 19.56 |
|  | ST DEV | 165.76 | 4.35 | 41.98 | 3.79 | N/A | 3.19 | 223.32 | 3.18 | 68.12 | 3.67 | 24.81 | 4.45 |
| **CS 8** | MEAN | 291.73 | 24.51 | 141.58 | 24.17 | N/A | 23.45 | 108.58 | 24.33 | 246.49 | 25.50 | 168.79 | 24.49 |
|  | ST DEV | 251.01 | 4.49 | 29.63 | 5.04 | N/A | 3.31 | 102.48 | 3.95 | 199.73 | 4.20 | 31.91 | 3.83 |
| **CS 9** | MEAN | 208.58 | 24.87 | 85.24 | 23.07 | N/A | 24.79 | 101.04 | 21.72 | 174.27 | 25.51 | 217.00 | 24.19 |
|  | ST DEV | 141.40 | 3.50 | 66.48 | 4.89 | N/A | 3.56 | 16.70 | 4.03 | 89.31 | 3.93 | 23.38 | 3.78 |
| **CS 10** | MEAN | 178.06 | 21.91 | 118.35 | 22.75 | N/A | 21.79 | 129.02 | 22.25 | 147.82 | 23.82 | 217.20 | 20.53 |
|  | ST DEV | 156.22 | 6.31 | 33.12 | 5.58 | N/A | 5.08 | 21.70 | 3.90 | 45.17 | 5.01 | 66.15 | 5.90 |

*Table 9. Normalized value of peak of the envelope and its position within the star descent cycle in analysed subjects, presented as mean and standard deviation of all trials per subject.*

| **Subject** |  | **RF PoE % DOWN** | **RF PPoE DOWN** | **TA PoE % DOWN** | **TA PPoE DOWN** | **PL PoE % DOWN** | **PL PPoE DOWN** | **GL PoE % DOWN** | **GL PPoE DOWN** | **MG PoE % DOWN** | **MG PPoE DOWN** | **EDC PoE % DOWN** | **EDC PPoE DOWN** |
| --- | --- | --- | --- | --- | --- | --- | --- | --- | --- | --- | --- | --- | --- |
| **DNS 1** | MEAN | 215.07 | 82.83 | 373.52 | 81.89 | 151.96 | 81.18 | 113.10 | 81.93 | 182.22 | 81.77 | 203.84 | 82.26 |
|  | ST DEV | 70.93 | 3.37 | 190.82 | 3.04 | 22.34 | 3.73 | 51.68 | 3.69 | 77.89 | 3.34 | 20.89 | 4.22 |
| **DNS 2** | MEAN | 326.96 | 81.77 | 609.11 | 79.56 | 818.12 | 79.86 | 249.79 | 80.93 | 148.97 | 79.63 | 490.91 | 80.25 |
|  | ST DEV | 62.78 | 0.44 | 134.25 | 1.53 | 201.25 | 1.19 | 34.11 | 0.55 | 22.58 | 0.55 | 386.54 | 1.64 |
| **DNS 3** | MEAN | 374.86 | 84.80 | 608.08 | 82.22 | 489.28 | 85.39 | 390.47 | 85.13 | 199.75 | 84.90 | 243.52 | 82.66 |
|  | ST DEV | 87.63 | 3.01 | 227.11 | 3.75 | 227.88 | 4.03 | 142.16 | 4.47 | 20.63 | 3.47 | 122.10 | 3.45 |
| **DNS 4** | MEAN | N/A | N/A | N/A | N/A | N/A | N/A | N/A | N/A | N/A | N/A | N/A | N/A |
|  | ST DEV | N/A | N/A | N/A | N/A | N/A | N/A | N/A | N/A | N/A | N/A | N/A | N/A |
| **DNS 5** | MEAN | 404.52 | 69.07 | 906.53 | 68.91 | 741.97 | 70.63 | 540.53 | 70.94 | 618.78 | 70.92 | 588.28 | 70.61 |
|  | ST DEV | 77.25 | 4.82 | 733.85 | 0.97 | 90.77 | 2.66 | 265.48 | 2.96 | 258.35 | 2.95 | 451.78 | 2.63 |
| **DNS 6** | MEAN | 129.10 | 71.12 | 389.12 | 72.05 | 246.58 | 71.37 | 88.44 | 71.24 | 212.11 | 73.02 | 95.79 | 72.12 |
|  | ST DEV | 36.81 | 3.53 | 39.61 | 4.38 | 54.68 | 3.32 | 22.02 | 3.70 | 94.81 | 3.31 | 35.82 | 3.58 |
| **DNS 7** | MEAN | N/A | N/A | N/A | N/A | N/A | N/A | N/A | N/A | N/A | N/A | N/A | N/A |
|  | ST DEV | N/A | N/A | N/A | N/A | N/A | N/A | N/A | N/A | N/A | N/A | N/A | N/A |
| **DNS 8** | MEAN | N/A | N/A | N/A | N/A | N/A | N/A | N/A | N/A | N/A | N/A | N/A | N/A |
|  | ST DEV | N/A | N/A | N/A | N/A | N/A | N/A | N/A | N/A | N/A | N/A | N/A | N/A |
| **DNS 9** | MEAN | 65.31 | 67.82 | 706.22 | 69.21 | 312.56 | 66.86 | 78.80 | 69.68 | 254.84 | 67.48 | 422.47 | 70.10 |
|  | ST DEV | 34.35 | 5.38 | 199.81 | 3.29 | 78.55 | 4.55 | 10.15 | 4.52 | 139.44 | 3.29 | 319.38 | 4.54 |
| **DS 1** | MEAN | 321.82 | 73.55 | N/A | 72.03 | 280.10 | 73.51 | 296.40 | 75.66 | 198.24 | 67.33 | 258.95 | 72.12 |
|  | ST DEV | 113.81 | 5.26 | N/A | 2.30 | 48.56 | 5.28 | 28.36 | 3.39 | 33.10 | 19.54 | 33.57 | 2.40 |
| **DS 2** | MEAN | 303.22 | 67.09 | N/A | 66.02 | 155.05 | 58.48 | 94.14 | 65.56 | 113.27 | 64.77 | 145.54 | 66.28 |
|  | ST DEV | 98.65 | 3.72 | N/A | 2.95 | 77.87 | 14.37 | 88.35 | 4.04 | 23.29 | 3.26 | 37.92 | 3.51 |
| **DS 3** | MEAN | 202.21 | 82.46 | N/A | 81.75 | 199.00 | 69.64 | 170.90 | 82.50 | 72.17 | 82.59 | 145.87 | 82.29 |
|  | ST DEV | 48.34 | 6.27 | N/A | 5.95 | 93.32 | 26.66 | 23.87 | 6.27 | 19.88 | 6.28 | 73.00 | 6.35 |
| **DS 4** | MEAN | 306.03 | 83.34 | N/A | 82.32 | 171.34 | 73.52 | 86.91 | 73.44 | 35.30 | 83.00 | 324.27 | 82.27 |
|  | ST DEV | 167.92 | 3.87 | N/A | 4.51 | 108.47 | 18.27 | 72.77 | 19.31 | 12.75 | 4.08 | 68.30 | 4.46 |
| **DS 5** | MEAN | 398.60 | 79.69 | N/A | 77.51 | 319.57 | 80.30 | 210.69 | 79.29 | 128.93 | 80.62 | 545.13 | 77.52 |
|  | ST DEV | 75.60 | 2.80 | N/A | 2.22 | 200.09 | 2.82 | 98.31 | 3.96 | 151.33 | 3.22 | 410.50 | 2.21 |
| **DS 6** | MEAN | 178.51 | 73.85 | N/A | 71.01 | 246.05 | 74.65 | 313.17 | 75.10 | 133.56 | 74.06 | 221.32 | 72.13 |
|  | ST DEV | 63.23 | 4.54 | N/A | 3.19 | 120.01 | 4.75 | 71.99 | 4.74 | 24.79 | 5.47 | 61.05 | 3.11 |
| **DS 7** | MEAN | 333.83 | 83.87 | N/A | 82.08 | 351.55 | 83.01 | 196.11 | 84.77 | 79.57 | 83.96 | 299.31 | 83.16 |
|  | ST DEV | 268.88 | 4.44 | N/A | 5.77 | 171.25 | 4.39 | 27.56 | 4.01 | 13.02 | 3.26 | 110.57 | 4.19 |
| **DS 8** | MEAN | 303.27 | 77.04 | N/A | 75.91 | 409.95 | 77.37 | 548.83 | 77.85 | 111.48 | 74.88 | 292.51 | 76.14 |
|  | ST DEV | 175.12 | 6.52 | N/A | 7.06 | 181.69 | 7.04 | 307.34 | 5.81 | 32.91 | 5.61 | 59.03 | 5.93 |
| **DS 9** | MEAN | 163.38 | 71.07 | N/A | 71.43 | 297.07 | 72.79 | 172.55 | 72.68 | 124.25 | 73.16 | 407.44 | 72.81 |
|  | ST DEV | 132.48 | 10.83 | N/A | 10.44 | 21.46 | 12.06 | 11.22 | 11.66 | 32.61 | 11.68 | 167.15 | 12.04 |
| **CS 1** | MEAN | N/A | N/A | N/A | N/A | N/A | N/A | N/A | N/A | N/A | N/A | N/A | N/A |
|  | ST DEV | N/A | N/A | N/A | N/A | N/A | N/A | N/A | N/A | N/A | N/A | N/A | N/A |
| **CS 2** | MEAN | N/A | N/A | N/A | N/A | N/A | N/A | N/A | N/A | N/A | N/A | N/A | N/A |
|  | ST DEV | N/A | N/A | N/A | N/A | N/A | N/A | N/A | N/A | N/A | N/A | N/A | N/A |
| **CS 3** | MEAN | 196.78 | 76.43 | 332.34 | 74.93 | 239.65 | 76.30 | 273.29 | 77.63 | 260.93 | 77.70 | 308.07 | 75.00 |
|  | ST DEV | 23.29 | 4.41 | 67.26 | 2.60 | 53.74 | 4.82 | 28.64 | 4.11 | 43.93 | 4.25 | 116.52 | 4.04 |
| **CS 4** | MEAN | 213.08 | 76.05 | 299.01 | 76.41 | 75.03 | 74.69 | 211.36 | 74.99 | 119.61 | 76.61 | 208.41 | 76.82 |
|  | ST DEV | 46.35 | 4.73 | 84.98 | 3.77 | 21.02 | 5.30 | 95.37 | 6.25 | 29.19 | 5.10 | 73.38 | 3.88 |
| **CS 5** | MEAN | 134.33 | 79.54 | 483.06 | 79.30 | 86.39 | 82.43 | 95.01 | 82.12 | 117.38 | 82.20 | 207.99 | 71.02 |
|  | ST DEV | 81.89 | 4.17 | 168.32 | 3.00 | 20.66 | 5.17 | 14.08 | 4.94 | 21.82 | 5.01 | 86.48 | 24.65 |
| **CS 6** | MEAN | 165.50 | 78.43 | 240.58 | 79.05 | 190.03 | 76.13 | 90.65 | 78.69 | 194.21 | 81.14 | 265.93 | 76.17 |
|  | ST DEV | 39.77 | 4.19 | 92.49 | 8.97 | 63.02 | 3.94 | 39.87 | 5.67 | 63.03 | 4.08 | 74.87 | 3.99 |
| **CS 7** | MEAN | 161.20 | 80.45 | 373.48 | 80.21 | 161.98 | 79.51 | 245.22 | 81.53 | 78.74 | 80.17 | 225.91 | 80.31 |
|  | ST DEV | 92.64 | 5.32 | 123.66 | 4.16 | 46.48 | 3.61 | 124.65 | 5.84 | 29.29 | 5.42 | 47.25 | 4.26 |
| **CS 8** | MEAN | 193.29 | 79.60 | 239.56 | 79.17 | 47.77 | 79.92 | 231.76 | 78.63 | 118.04 | 80.54 | 194.10 | 79.65 |
|  | ST DEV | 95.42 | 4.15 | 126.06 | 3.69 | 40.78 | 2.97 | 93.91 | 3.60 | 30.22 | 3.90 | 32.58 | 2.32 |
| **CS 9** | MEAN | 181.43 | 80.67 | 388.04 | 81.17 | 180.52 | 84.08 | 108.43 | 81.29 | 158.27 | 84.90 | 299.56 | 83.25 |
|  | ST DEV | 68.76 | 4.70 | 115.25 | 3.39 | 138.13 | 4.38 | 64.00 | 5.54 | 34.11 | 4.27 | 188.08 | 4.26 |
| **CS 10** | MEAN | 129.76 | 77.18 | 540.95 | 76.04 | 240.08 | 79.58 | 113.11 | 79.21 | 158.24 | 79.94 | 356.56 | 75.97 |
|  | ST DEV | 37.63 | 7.86 | 403.94 | 7.17 | 134.86 | 6.15 | 45.02 | 6.03 | 33.10 | 6.53 | 274.22 | 5.30 |


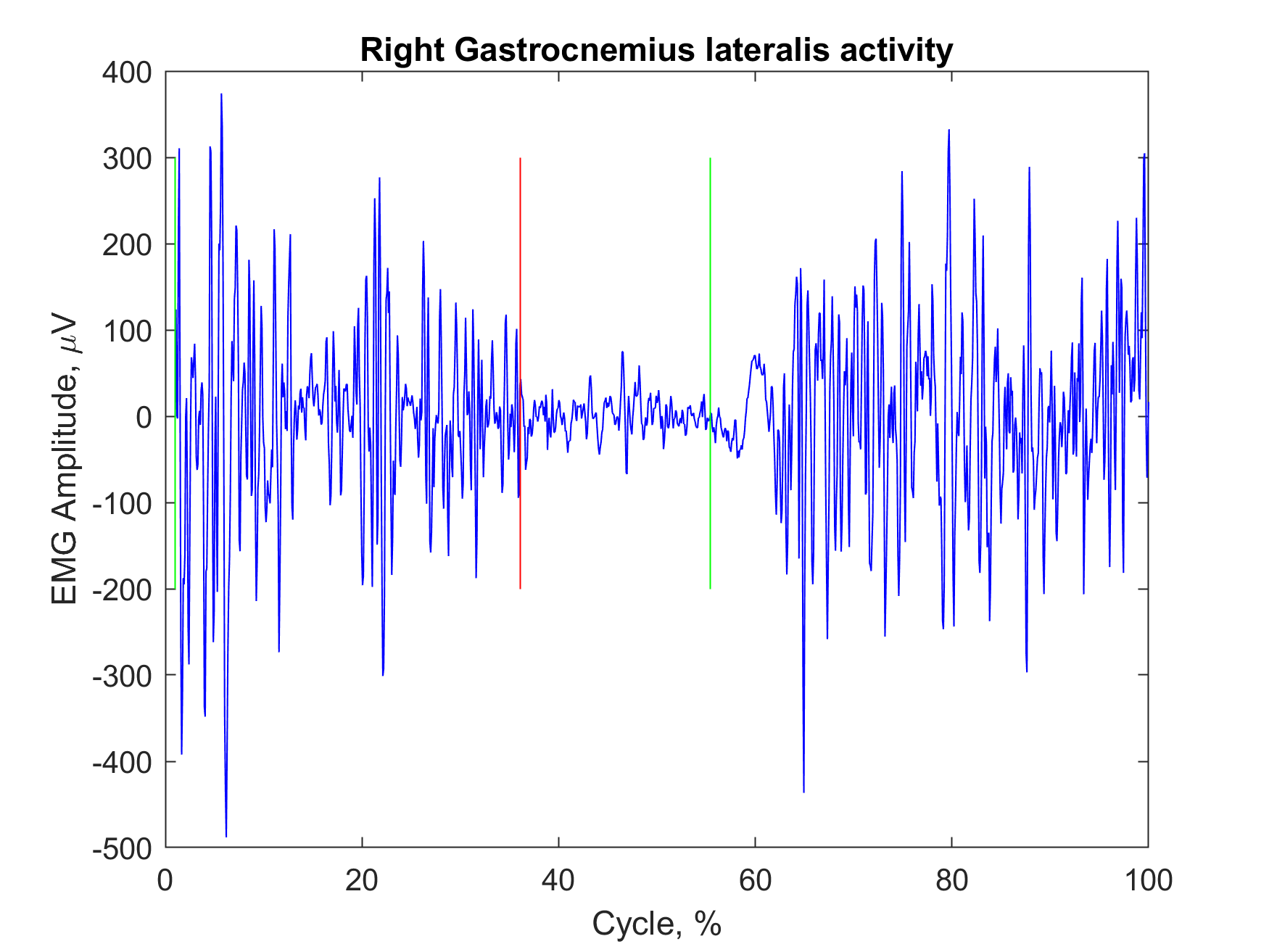


Figure 6. Muscle activity during gait in CS subject.


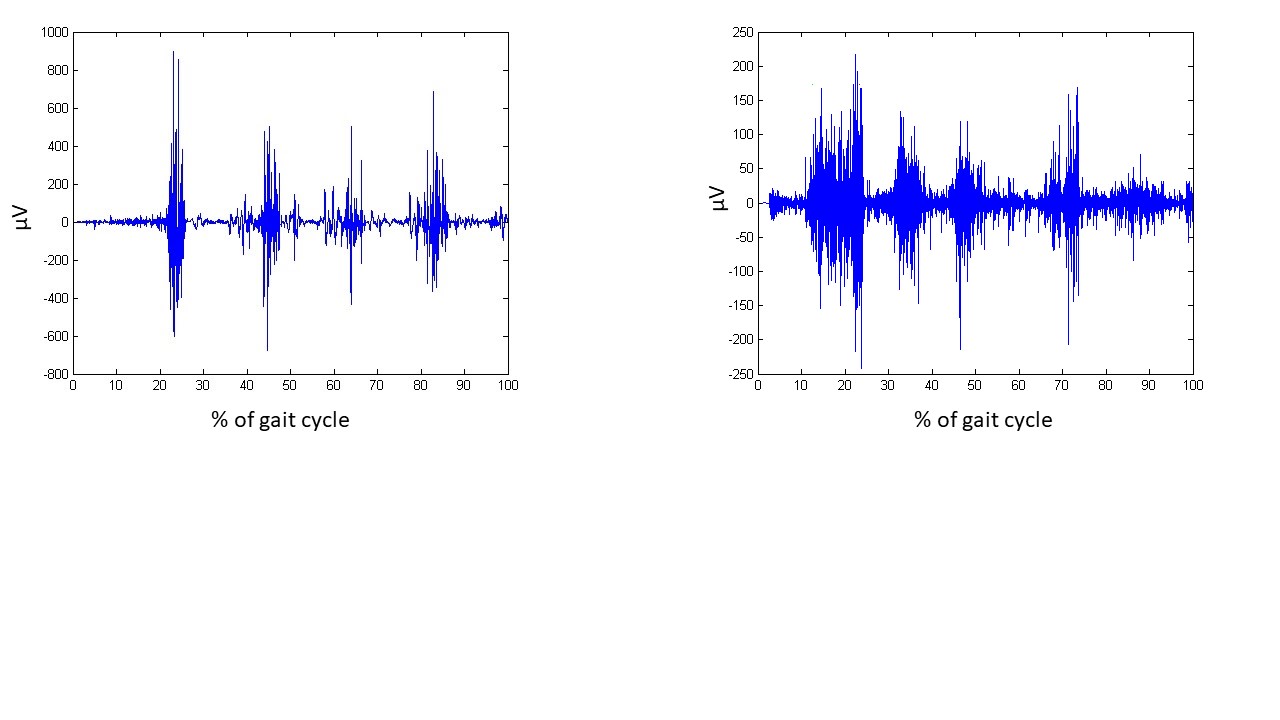


Figure 7. Muscle activity during stair negotiation in CS subjects.


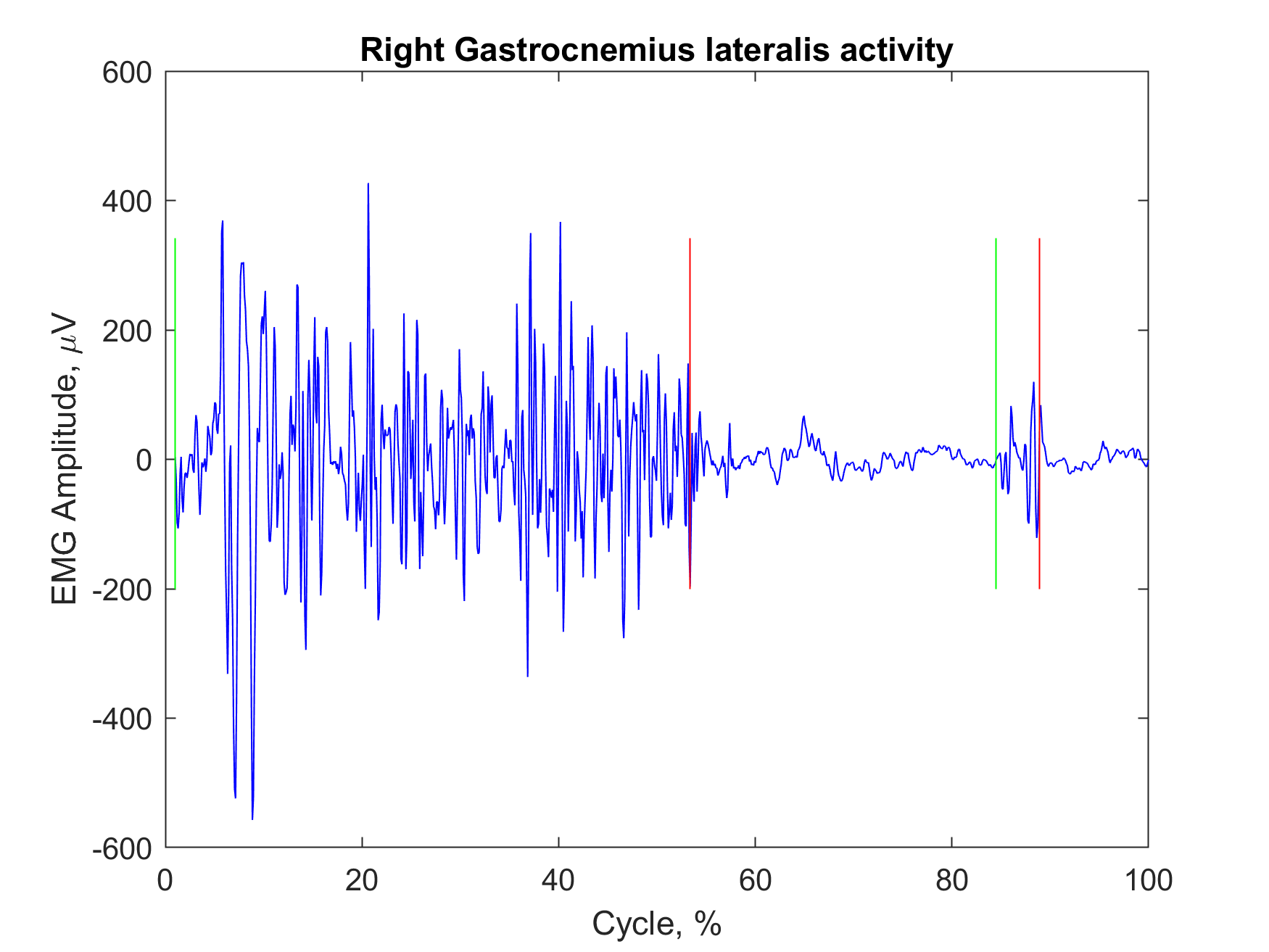


Figure 8. Muscle activity during gait in DNS subjects.


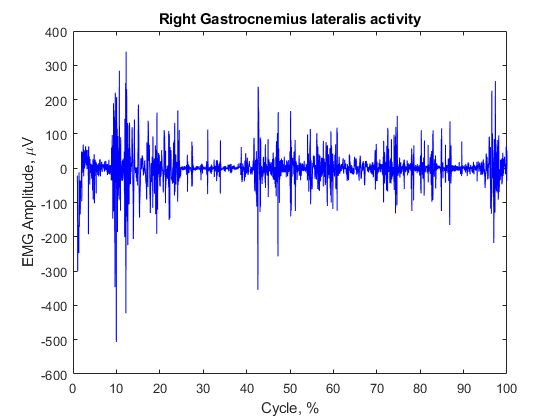


Figure 9. Muscle activity during stair negotiation in DNS subjects.


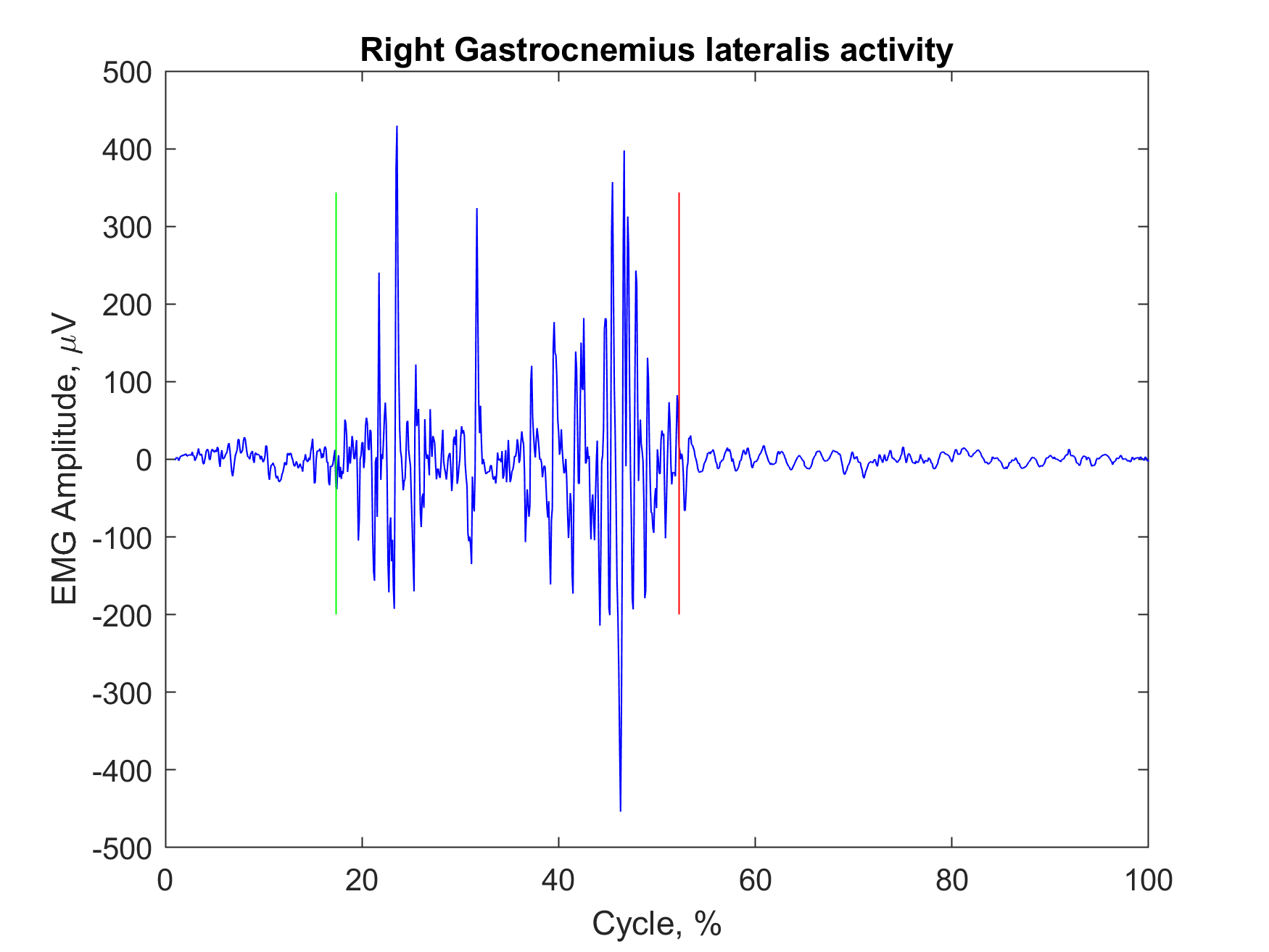


Figure 10. Muscle activity during gait in DS subjects.


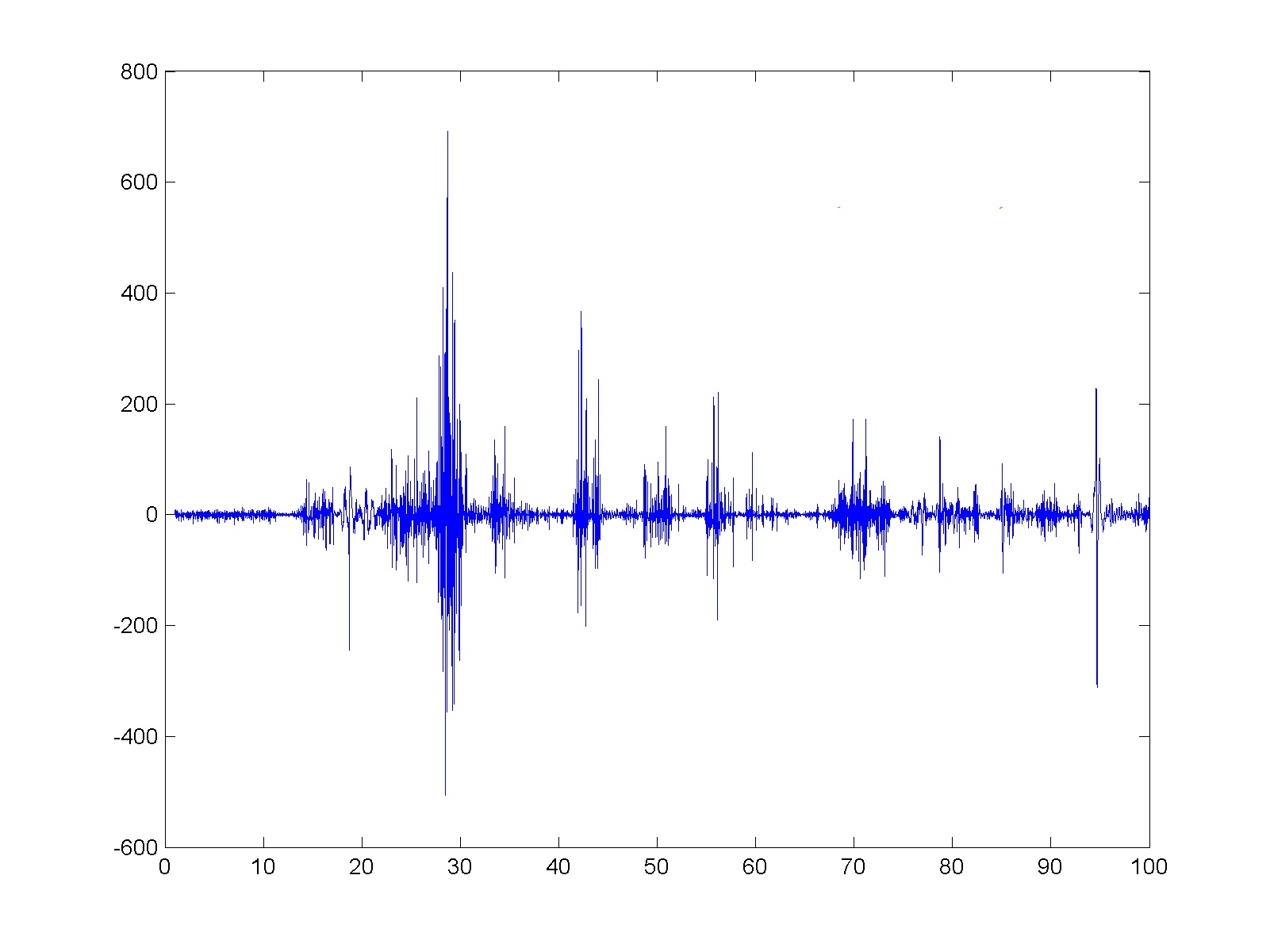


Figure 11. Muscle activity during stair climbing in DS subject.
